# Low Cost Photonic Sensor for in-Line Oil Quality Monitoring: Methodological Development Process towards Uncertainty Mitigation

**DOI:** 10.3390/s18072015

**Published:** 2018-06-22

**Authors:** Patricia Lopez, Jon Mabe, Guillermo Miró, Leire Etxeberria

**Affiliations:** 1 IK4-Tekniker, 20600 Eibar, Spain; jon.mabe@tekniker.es; 2 atten2 Advanced Monitoring Technologies, 20600 Eibar, Spain; gmiro@atten2.com; 3 Mondragon Unibertsitatea, 20600 Eibar, Spain; letxeberria@mondragon.edu

**Keywords:** optoelectronic and photonic sensors, optical sensors, lubricating oils, hydraulic fluids, maintenance, condition monitoring, product design, product development, simulation, prototypes

## Abstract

Lubricant and hydraulic fluid ageing impacts the performance of the machines, gears, transmissions or automatisms where they are being used. This manuscript describes the work accomplished for bringing an innovative measurement concept for analysing the physical- chemical properties of these fluids, to a real industrial product ready to be integrated into different industrial equipment. The steps taken to deal with uncertainties and evolving requirements while progressing in the sensor development are described, covering the stages of theoretical formulation of the problem, optical and fluidic simulations, sensor prototype development and tests. The sensor working principle is based on a combination of transmittance and diffuse reflectance photonic inspection of the fluid sample that is collected in a micro-cavity through a standard hydraulic fitting. Photonics, electronics, micro-mechanics, fluidics, data processing and analysis has been merged with a deep knowledge in the lubricant degradation process to develop a sensor solution that is able to measure the Oil Degradation Index, Oil Oxidation, Acid Number, Ruler and Membrane Patch Colorimetry data from an in-service lubricating oil sample. The photonic micro sensor presented here offers a powerful tool that operates directly immersed in the fluid, at an economic cost and compacted size for in-line oil degradation monitoring.

## 1. Introduction

Oil degradation could be defined as the process by which the performance of the fluid decreases the impact on its ability to perform basic lubricant functions such as lubrication, cooling, cleaning, protection or sealing [1]. Processes such as oxidation, nitration, temperature rise, external contamination, shearing, corrosive ambient, additive depletion, etc. are the most important factors contributing to and accelerating the oil degradation process [2]. Therefore, lubricant and hydraulic fluid ageing impacts the performance of the machines, gears, transmissions or automatisms where they are being used [3,4], and often the degradation of the lubricant properties is the cause of downtimes and dramatic failures [5,6]. Consequently, the periodic change of these oils is a basic part of the maintenance program of the infrastructure operators [7], with an important impact on the O&M (Operation and Maintenance) cost, and different research and commercial efforts have been fostered trying to migrate to predictive maintenance approaches with the aim of minimizing oil change while assuring the safe operation of the infrastructure [8]. In this context, the number of in-line sensors installed in industrial equipment for monitoring the status of the fluids has been importantly increased [9,10,11].

Additionally, in the last couple of years, different trends of the predictive maintenance approach have merged with the Internet of Things (IoT) [12,13]. In this context, the smart sensors are considered a key factor in meeting the promise of unprecedented advancements in automation, efficiency and flexibility, together with an expected dramatic saving in manufacturing, operation and maintenance costs. Focusing on industrial scenarios, in the Industry 4.0 or the Industrial Internet of Things (IIoT) approaches, the smart sensors are considered as the real enablers for a true industrial digitalization, since these sensors become the unique data sources for all the services and big data applications [14,15]. In this context, large companies are not only focusing on the higher tiers of the digitalization of industry [16], but also, they are shifting to generate novel sensor technologies that meet the expectations of the IIoT in terms of integrability, connectivity, intelligence, flexibility, rugged and reliable operation and indeed, cost [17,18]. This eagerness for pervasive motorization within industrial environments represents a paramount opportunity for sensor manufacturers, with different applications pulling for the in-place and real time measurement solutions. 

**Figure 1:**
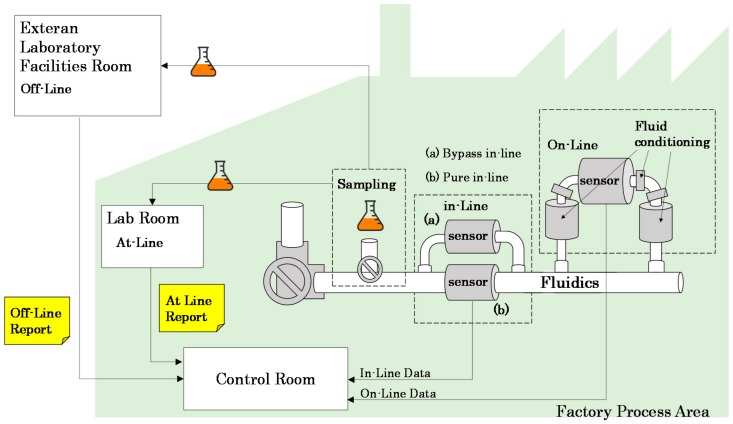
Diagram depicting the differences between the fluidic process analysis approaches [21].

Table 1 summarizes possible application scenarios where these kinds of sensors are required. Several types of machines classified by sector and some of its main lubrication technical characteristics are presented. In addition, the severity of lubrication failures and their possible consequences are indicated. Most of these sectors require large moving machinery in which sensors can be installed with a bypass connection. However, it is worth highlighting sectors such as automotive or manufacturing with small motors, robots, cranes, or small mechanical transmission systems, where making a bypass connection is not easy [19]. In addition, a bypass connection could complicate installation and require bulky settings and extra investment for sensor in-line operation, due to requirements for fluid conditioning, etc. This situation leads to the necessity of easily integrable and compact sensor solutions, like bolt stem or plug-in approaches (see Figure 1, pure in-line connection (b)). Moreover, scenarios like production plants with thousands of mechatronic lubricated systems to be monitored require economic solutions [20]. These use cases are clear examples demanding a trade-off solution between an immediate availability and reliability of information, and integrability, compactness and economic cost, which is the ideal scenario for solutions like the one presented in this paper. 

However, meeting the already mentioned requirements in terms of sensor performance, compactness, unitary cost, reliability, etc., but also market and economic objectives poses a considerable technical and organizational challenge for any company. Management of uncertain and evolving specifications, continuous risk assessment, synchronizing of multidisciplinary working teams (e.g., micromechanics, optics, electronics, fluidics, etc.) are common challenges to be solved in the field of sensor development [21]. In addition, increasing importance of time to market (TTM) and project costs reduction in order to be competitive [22,23], and the complexity inherent to the technological bases of the sensor’s scientific measurement principles need to be managed efficiently throughout the whole development process of the sensor product.

In this context, the work presented in this paper describes a pluggable photonic sensor for in-line monitoring lubricant quality, and methodological development process followed for bringing innovative sensor idea into a fully operative, reliable, compact and cost-effective sensor system. 

### 1.1. Lubricant Quality Parameters and Sensors

First introduced in the late forties [24], the laboratory analysis of lubricating oil samples looking for evidence of ageing or degradation has been continuously evolving to offer a more accurate diagnosis about the remaining life time of the lubricants, or about any latent or imminent failure in the equipment or processes where they are being used. These laboratory analyses are nowadays standardized as a set of parameters describing the quality of an oil, which some of them are described below [25,26]:

1. Acid Number (AN), is a measurement of oil acidity and it is considered a key indicator that may reflects oil quality deterioration due to chemical reactions, oxidation, incorrect oils, additive depletion and contamination. ASTM (American Society for Testing and Materials) D974 and ASTM D664 methods are the current industry standard methods for measuring the acid number [27,28,29].
2. Remaining Useful Life Evaluation Routine (RULER) by Linear Sweep Voltammetry is a test method to determine the remaining useful life of oil by measuring remaining antioxidant additives. These additives are responsible for slowing down the oil degradation process, and therefore remaining additives level may indicate the oil degradation condition. ASTM D6971 and ASTM D6810 are approved test methods specified by ASTM [30,31].
3. Fourier Transform Infrared Spectroscopy (FTIR) evaluates the oil’s components at a molecular level. The spectrum is used as a fingerprint of various components and it is compared with fresh oil reference. High concentrations of degradation products, such as oxidation, nitration, and sulphation, and low concentrations of oxidation inhibitors indicate oil degradation. However, FTIR interpretation is complex and some additive components usually mask the critical antioxidant region. The ASTM E2412 test method is the approved standard practice for lubricants condition monitoring by FTIR spectrometry specified by ASTM [32].
4. **Viscosity**. Lubricants need to have a specific viscosity to give correct operation in the equipment where they are being used. Viscosity change as the oil oxidizes, whereby this is an important parameter to measure when the oil condition is evaluated. ASTM D445 the standard test method for viscosity approved by ASTM [33].
5. ASTM Color Scale (ASTM D500), is a method that visually determines the colour of petroleum products such as lubricating oils, waxes, and diesel fuel oils. If the colour range of a specific oil is known, a variation outside this may indicate contamination. Colour can also be used as an indication of the degree of refinement of the material [34].
6. Membrane Patch Colorimetry (MPC) is a method for analysing the insoluble contaminants in lubricants with spectral analysis. With MPC, a direct correlation is made from the colour and intensity of the insoluble to lubricant oil degradation. The method identifies soft contaminants, which are directly associated with oil degradation. Larger hard contaminants unrelated to oil degradation do not influence the test results much. ASTM D7843 is the approved standard test methods specified by ASTM [35,36].

As mentioned earlier, huge efforts are being made to migrate these laboratory measurements right to the field. Several scientific references are found related to sensors for real-time oil quality monitoring. Those sensors use different measurement principles to provide the oil quality condition that can be related with previously listed parameters. As an example, Torres et al. [37] propose a low-cost sensor solution based on the measurement of the complex permittivity of the lubricant. Rauscher et al. [38] use a non-dispersive infrared spectroscopy measuring principle to monitor the oxidation, water content, and acid number of the lubricating oil in automotive and industrial gearboxes. Han et al. present a cylinder capacitive sensor to detect changes in the lubrication oil condition of an engine lubrication system [39]. Moreover, sensors based on other electrical properties measurements have been also studied, such as Chen et al. [40], Shinde et al. [41] and Riziotis et al. [42].

In the context of plug-in in-line oil condition sensors, diverse scientific references, patents and commercial sensor have also been found. Table 2 lists some of the current commercial sensors. Those sensors provide an easily integrable and compact solutions to avoid hydraulic bypass connection and comply with the integrability requirements presented before. The table also shows an estimation regarding integrability, price and size characteristics for each of the presented commercial sensors.

Narrowing down to solutions based on the photonics, additional scientific references and patents are found. These solutions also provide an easily integrable hydraulic fitting compatible with the plug-in sensors to operate directly immersed in the fluid to be monitored. For example, Welling et al. [43] patented in-situ fluid analysis sensor bolt for fluids used in machinery such as engines, compressors or hydraulic devices. An infrared source of radiation is configured to direct the radiation through the flow path containing the fluid sample, and a detector detects the radiation passing through the path. The patent does not describe the correlation between obtained measure and the oil degradation condition. On the other hand, Miranda et al. [44] patent a compact colourimeter that can be submerged in liquids for monitoring the colour of liquid samples according to CIELAB standards. The device consists of a colour charge-coupled device (CCD) video camera, two lighting systems to measure by reflectance (opaque samples) or transmittance (translucent samples) and the necessary optics for capturing the light that interacts with the monitored sample. As in the previous patent, the system measures the colour of a fluid sample, but it does not correlate the measured colour to a fluid condition. Finally, Ossia et al. [45] present an optical sensor for synthetic hydraulic oils condition monitoring based on oil colour change compared to fresh oil sample. The device integrates a white light emitter that illuminates the oil sample, a colour sensor that receives the light that passes through the oil and a feedback photodiode to stabilize the optical power of the emitter. Based on measured oil colour a total contamination index is calculated in an external microcontroller. 

However, described photonic measurement principles are based on light transmittance through the fluid sample, from the light emitter to the detector. This optical setting requires a high optomechanical accurateness to guarantee specified distances and alignment of the photonic and optical elements [46,47]. This fact adds complexity to sensors manufacturing process and consequently manufacturing and sensor cost increases. In addition, as most of the referenced approaches include protected intellectual property, their commercial use for new developments is, evidently, restricted. These limitations encourage us to research on new sensor concept approaches to face these technical and commercial challenges.

### 1.2. Transmittance and Diffuse Reflectance Measurement Principle

**Figure 2:**
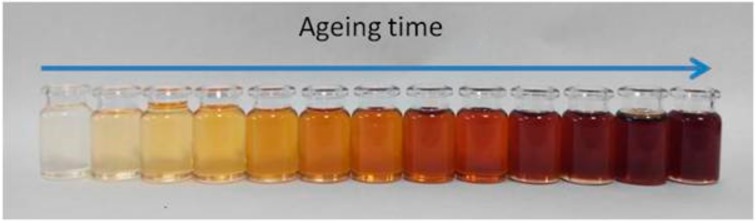
Change in the lubricant visible spectrum due to degradation.

This paper describes the development of a photonic sensor to determine oil degradation level based on the colour variation of an oil sample compared to fresh oil. On one hand, there is an important previous work done on correlating the physical-chemical parameters of the lubricant oil with colour variations over ageing time (see Figure 2) [48,49,50,51,52]. Based on this scientific background, the proposed sensor will embed algorithms [52] to provide a general oil degradation index combining the previously defined laboratory parameters (AN, RULER, FTIR, etc.). 

**Figure 3:**
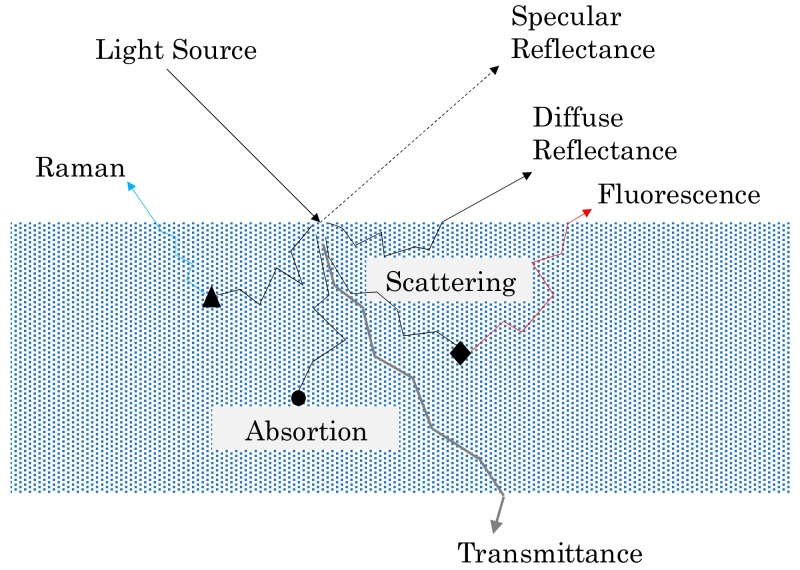
Summary of the different processes after the interaction between an incident light ray and a sample material.

On the other hand, there is also previous experience in photonic sensors development based on light matter interaction for accomplishing measurements about chemical and physical parameters in fluids, not only for industrial but also for food sectors [52,53,54,55,56,57,58,59,60]. Figure 3 describes the most important effects and phenomena (reflection, absorption, diffraction, scattering, etc.) happening when the light radiation enters a matter, due to sample chemical and physical properties [61,62,63]. The study of the modifications that happen to the emitted light is the base of the aforementioned photonic sensors. In the context of lubricant oils, the change in the colour over ageing time due to physical-chemical changes in the fluid is related to light-matter interactions. So, the collection and the subsequent analysis of light that comes out the matter provides relevant information about fluid characteristics.

**Figure 4:**
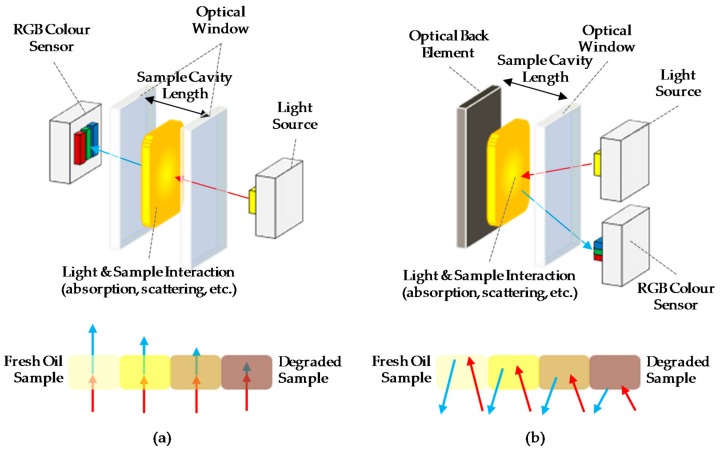
(**a**) Transmission and (**b**) Reflection approaches for fluid sample RGB absorbance measurement.

In this context, this paper presents an innovative sensor concept approach that combines previous experience and knowledge in both the photonic sensor development and the correlation of the physical-chemical variables of the lubricant oil with the change in colour over ageing time. The starting point of the sensor proposed in the article is the OilHealth^®^ sensor, previously developed for atten2 Advances Monitoring Technologies (Eibar, Spain). OilHealth^®^ is an industrial in-line photonic sensor for real-time oil quality monitoring, based on transmittance measurement principle (see Figure 4a) [8,52,53]. A white light beam goes through the oil inside the device sample cavity and an RGB colour sensor collects the light transmitted through the oil. The obtained RGB value is processed by embedded algorithms that compare it to the RGB measurement of the corresponding fresh oil sample. The algorithm result provides an oil degradation index (OD) that indicates the total degradation percentage of the oil sample. Those algorithms are based on previously established correlations between physical-chemical variables of the lubricant oil and sample colour change regarding fresh oil [52]. To manage the sample cavity filling and oil sample renewal, OilHealth^®^ requires a bypass hydraulic connection and integrates a sample conditioning hydraulic subsystem, which represents an important drawback for complying with the requirements in terms of integrability, compactness and unit cost, specified before.

Therefore, the idea to develop an innovative photonic sensor based on transmittance and diffuse reflectance measurement principle is outlined. Thus, patent infringement is avoided, and the sensor set up is simplified. As it is shown in Figure 4b, in this photonic configuration, the light beam passes through the fluid sample, is reflected in an optical back element and passes back through the fluid sample, which is a clear innovation from the referenced systems. Then, the RGB colour sensor placed beside the light source collects the light that is not absorbed by the fluid and is transmitted and reflected. The optical back element acts like a virtual light emitter directing the light radiation again through the fluid sample to the optical receiver. Finally, as in OilHealth^®^, the colour (RGB) change due to changes in the light absorbance is used to calculate the oil degradation index (OD). 

As shown in Figure 4, the main design difference between OilHealth^®^ and the new approach is that the optical transmitter and receiver are placed in the same mounting plane, which constitutes a simplification of the optomechanical and optoelectronic design. This optical element setting has the following advantages over OilHealth^®^ or similar sensors designs:

- Required optomechanical accuracy for optical elements is minimum.
- Fluidical-Mechanical subsystem redesign allows the sensor to operate directly immersed in the fluid, avoiding the necessity of a hydraulic conditioning subsystem required for a hydraulic bypass connection.

These advantages contribute to reduce manufacturing costs, allow the miniaturization of the system and facilitate its integration and installation in scenarios where OilHealth^®^ is not a suitable option. Therefore, this new sensor concept approach potentially meets previously identified requirements in terms of integrability, compactness and cost. 

However, the proposed sensor concept involves three important uncertainties that impact reliable sensor operation and hinder sensor development.

- Although both transmittance and reflectance approaches (see Figure 4) are conducted in the visible spectrum and the light that is not absorbed by the oil is collected by the detector, the light and fluid interaction process differ. This difference makes it impossible to determine at the beginning of the development the RGB absorbance measurement dynamic range that can be achieved with the proposed optical configuration. The RGB dynamic range is the key element of the development since it is directly related to the sensor’s oil degradation measurement resolution and the compatibility with different oil types that could be measured.
- The dependence between the temperature and some optical elements, such as, light emitters, receivers, etc., is known. For example, the white LEDs lose luminous intensity as temperature increases. Although the manufacturers of the optical elements provide this efficiency versus temperature dependency, the real impact of this effect in the RGB light absorbance measurement is uncertain. It is necessary to precisely analyse this dependency and guarantee light source stability since uncontrolled variations in the RGB light absorbance measurement can lead to an erroneous oil quality value.
- As the sensor operates immersed in the fluid, the filling of the sample cavity and renewal of the oil sample cannot be guaranteed by means of hydraulic actuators. In this context, there is an uncertainty regarding the ability of the fluidical-mechanical sensor design that ensures sample cavity filling, air evacuation and fluid sample renewal considering the different conditions of the sensor installation in the field (e.g., viscosity, temperature and pressure of the fluid). This is also an especially relevant point, because the presence of bubbles in the sample cavity, non-renewal of the oil sample, etc., can lead to an erroneous oil quality value.

In addition, the development aims to increase the amount of information provided by the sensor, related to oil degradation condition. Besides the Oil Degradation (OD) value, it is of great interest to provide more specific information such as aforementioned Acid Number (AN), Ruler, etc. parameters. The calculation of those parameters from the RGB absorbance measurement entail reformulating previously established correlations between the physical-chemical variables of the lubricant oil and sample colour change. These algorithms will be also integrated in the proposed sensor concept.

Finally, the following sections describe the work accomplished for bringing the presented photonic principle into a fully operative, reliable, easily integrable, compact and cost-effective industrial sensor for in-line monitoring lubricant quality. Section 2 presents the methodological process followed to face and solve identified uncertainties. Section 3 describes in detail the activities carried out during this process, including theoretical approaches, simulations and prototypes development. Section 4 shows obtained results and Section 5 concludes this article.

## 2. Materials and Methods 

This section presents the methodological procedure followed to support the development of the proposed sensor concept approach. The development of such systems entails technological, scientific and operational challenges [64,65] that derive from: (i) missing requirements, uncertainties and risks related to the generation of new products based on novel technologies and innovative ideas [66]; (ii) unforeseeable interactions among the system components intrinsic to system heterogeneity and complexity (e.g.,: software, hardware, chemistry, optic, mechanic, etc.) [67]; (iii) operation dependability and regulatory requirements imposed by critical domains such as industry or energy; and (iv) increasing importance of time to market and project costs reduction in order to be competitive [23].

To efficiently manage those challenges, a structured and iterative Design/Build/Measure development process has been followed. This process is based on a hybrid approach where ideas from New Product Development Processes as “Design for Six Sigma” [68], “Lean Startup” [69], “Open Innovation and Stage-Gate” [70,71] have been merged. Those approaches are mainly focused on shaping the system and identifying changes and deviations at relatively early development phases so the impact of required modifications, and consequently development cost and time, are minimized. The process is based on theoretical approaches, simulations and prototypes development as intermediate solutions to reach the final sensor system. These intermediate solutions favour exploring and testing, which is essential for uncertainties resolution and complete requirements (risks and limitations) specification.

First, based on customer and market requirements, functional, technical and design uncertainties involved in the proposed sensor solution, and mechanisms or activities to solve them are identified. The aim is to prioritize the resolution of uncertainties with a greater impact on the basic functionality of the system. Based on this information, a development activity plan is formulated to guide the development process. Every time an activity is completed, obtained results are evaluated and corresponding technical and design decisions are taken to successfully continue the development. This assessment should not only consider the activity results, but also customer feedback, new requirements, etc. In addition, if necessary, the activity plan is updated according to the conclusions of the assessment. Once all the activities are completed, a fully specified solution is obtained, without uncertainties and with identified limitations and risks. At this development point only the additional functionality of the system in terms of embedded algorithms, communications, etc. remains to be implemented. 

As presented in the previous section, the main uncertainties jeopardizing the development of the proposed sensor concept approach are related to the optical and fluidic subsystems and the corresponding impact on reliable sensor operation. To provide a reliable performance in terms of oil degradation condition monitoring, the RGB measurement must be significant to cover the widest range of oil types and degradation levels, and stable in operation conditions. However, as mentioned, the achievement of this expected performance involves several technical and design uncertainties summarized in Table 3. Additionally, this table displays the mechanisms (theoretical approaches, simulations and prototypes development) to face, mitigate and finally solve those uncertainties. 

**Figure 5:**
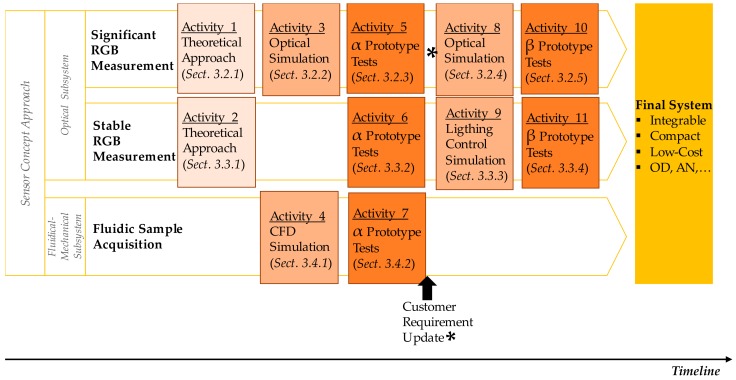
Structure of the sensor development process.

Next, those uncertainties and mechanisms are translated into the development activity plan presented in Figure 5. As explained before, the development process is structured in several activities to face and solve identified uncertainties, prioritizing the ones with greatest impact on sensor’s basic functionality. Those activities are mainly focused on optical theoretical approaches, optical and computational fluidic dynamics (CFD) simulations, and prototypes development and tests. This is an updateable process that could be modified if the results of the activities are unsuccessful or if the design changes and new activities are required. Or, as in this case, the customer considers new requirements that impact system design, and the development of new optical simulations and a second prototype are required. The execution of those activities is described in detail in Section 3.

## 3. Uncertainty Driven Sensor Design

This section describes the main building blocks of proposed sensor solution and activities carried out to solve technical, functional and technological uncertainties presented in the previous section. 

### 3.1. Sensor General Description and Prototypes

**Figure 6:**
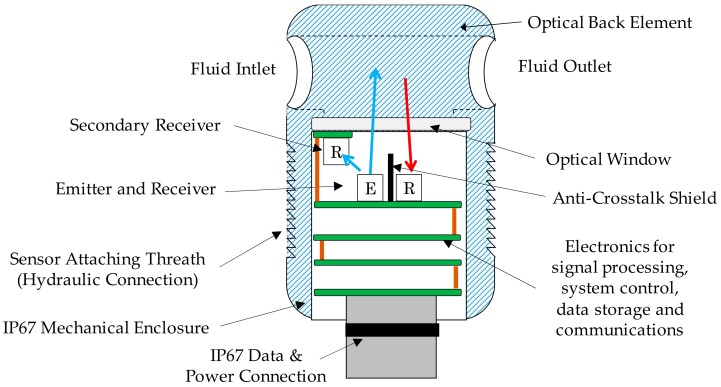
Component diagram of the sensor concept approach.

**Figure 7:**
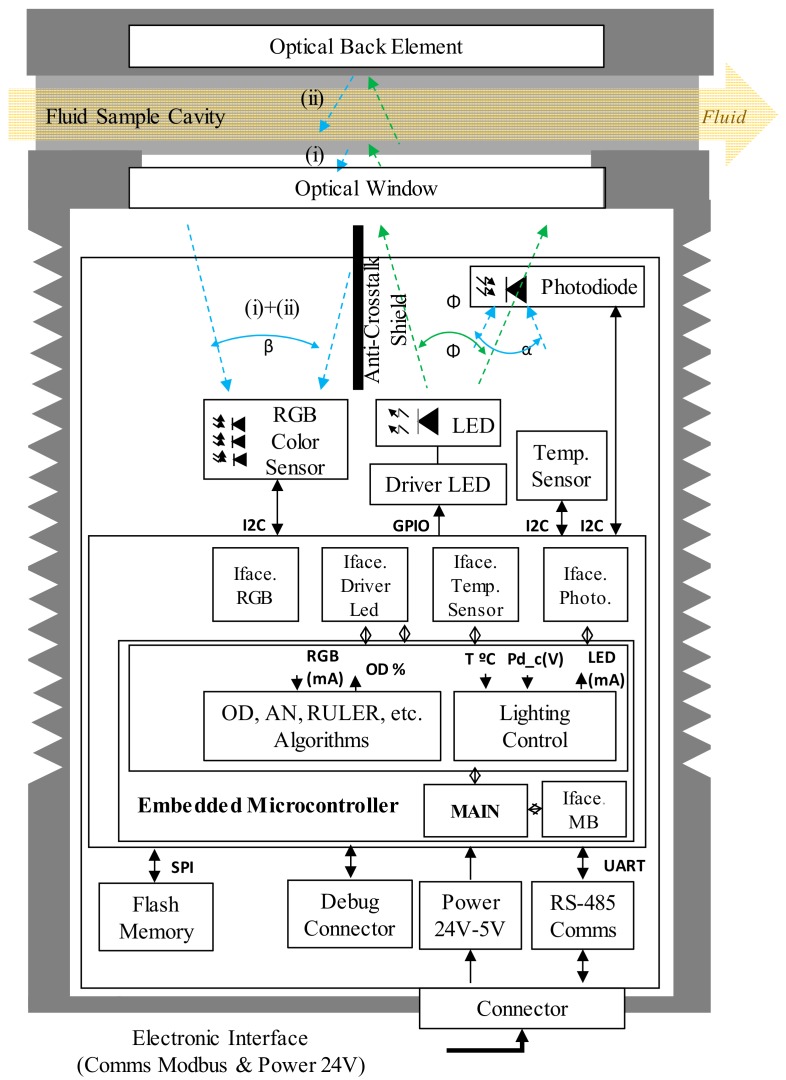
Hardware and software block diagram of the sensor concept approach.

**Figure 8:**
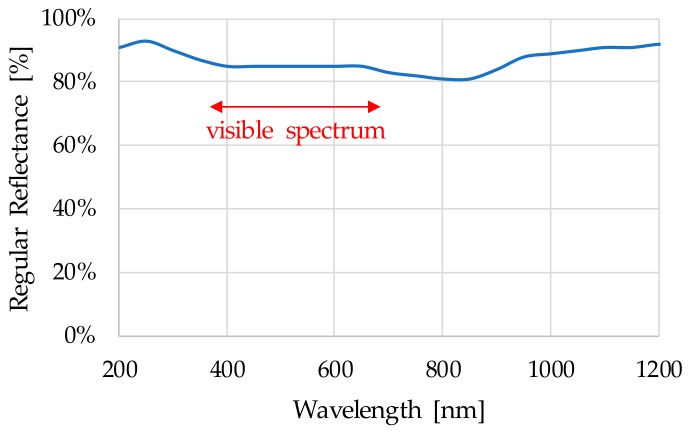
Polished aluminium reflectance spectrum.

The proposed sensor concept approach integrates different building blocks, including mechanic, fluidic, optic, electronic, embedded software, etc. As it is shown in Figure 6 and Figure 7, the fluidical-mechanical subsystem includes the fluidic sample cavity, the hydraulic connections or sensor attaching thread, and holds and positions the optics and electronics components. The optical subsystem integrates an RGB colour sensor (HAMAMATSU S11059-02T digital colour sensor, Hamamatsu Photonics, Hamamatsu City, Japan), a white LED lighting (CREE^®^ PLCC4 1 in 1 SMD LED CLA1A-WKW, CREE^®^, Research Triangle Park, NC, USA), an ambient light sensor (MAXIM MAX44009, Maxim Integrated, San Jose, CA, USA) acting as secondary receiver, a glass window (BK7 high quality optical glass) and the optical back element (polished aluminium) to confine the fluid within the fluidic sample cavity. The use of polished aluminium is proposed as it has an almost flat reflectance of 90% for visible spectra (see Figure 8). In addition, to avoid the direct radiation from the emitter to the receiver, an anti-crosstalk shield is placed between the light source and the RGB colour sensor. Finally, the sensor includes custom embedded electronics for lighting control and the execution of the oil degradation algorithms. As mentioned before, these algorithms are the result of previous work done on correlating the physical-chemical parameters of the lubricant oil with the colour variations over the ageing time [48,49,50,51,52]. The algorithms are directly implemented in the embedded electronics. 

**Figure 9:**
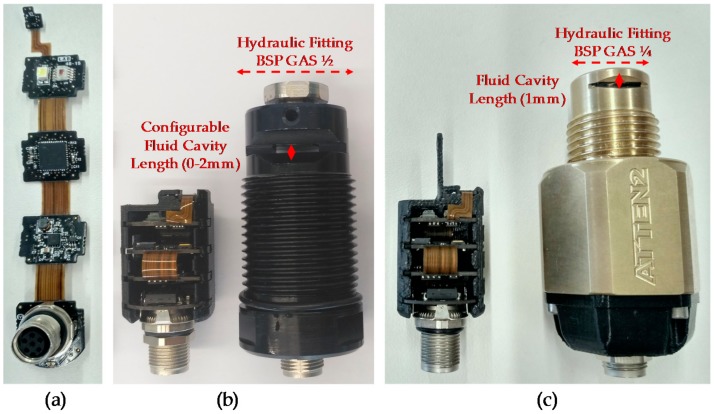
(**a**) Custom embedded electronics; (**b**) Sensor Alpha Prototype; (**c**) Sensor Beta Prototype.

Two prototypes have been developed to achieve desired final system. Alpha Prototype has a configurable sample cavity length to test different optical configurations and fluid pathlength, and determine the position that maximizes the RGB measurement dynamic range. Since theoretical approaches and simulations have not been determinant enough to define the optical configuration, this prototype has been developed. In addition, this prototype is used to verify the fluidical-mechanical subsystem and the compliance of the sample acquisition requirements. Then, a second prototype (Beta prototype) is developed, motivated by new customer requirement. This requirement specifies the necessity to reduce the hydraulic fitting size. This modification requires increasing the distance from the optical emitter and receiver to the sample cavity, so RGB measurement resolution could be affected. Beta prototype and corresponding optical simulation verify the feasibility of this innovative design. Figure 9 shows developed prototypes and remarks on their main differences.

Finally, as mentioned before, the following subsections describe the activities carried out to solve previously identified uncertainties and develop the devised sensor systems. The development process is divided into the development of the optical and the fluidical-mechanical subsystems, and includes the activities listed in Table 4: Section 3.2 and Section 3.3 describe the optical subsystem development. Those sections describe the activities 1, 2, 3, 5, 6, 8, 9, 10 and 11 defined in the activity plan, that are related to optical theoretical approaches, optical simulations, and tests with Alpha and Beta prototypes. In addition, Section 3.3 describes the development of the fluidical-mechanical subsystem. This part of the development is addresses by fluidic dynamic (CFD) simulations (activity 4 of the activity plan) and tests with Alpha prototype (activity 7). 

### 3.2. RGB Measurement Dynamic Range

This subsection presents the activities carried out to solve the technical, functional and design uncertainties of the optical subsystem, regarding the definition of the RGB measurement dynamic range. First, a theoretical approach is made to approximately determine the position and characteristics of the optical elements of the Alpha Prototype. Next, the proposed design is verified via simulation, and finally the prototype is validated with real oil samples. In addition, a second iteration of simulation and tests is performed to evaluate modification related to Beta Prototype.

#### 3.2.1. Theoretical Approach of the Alpha Prototype (Activity 1 of the Activity Plan)

**Figure 10:**
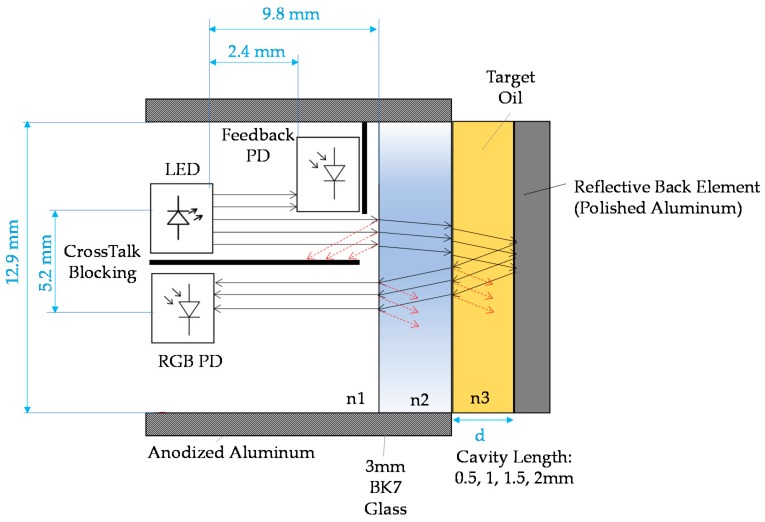
Basic setup and component characteristics of the chosen measurement principle.

This section describes the theoretical approximations made for describing the basic chemical and physical processes involved in the chosen measurement principle. First, the basic setup and components (see Figure 10) with their significant characteristics need to be defined, where optoelectronic components, materials and target fluid must be characterized as precisely as possible.

**Figure 11:**
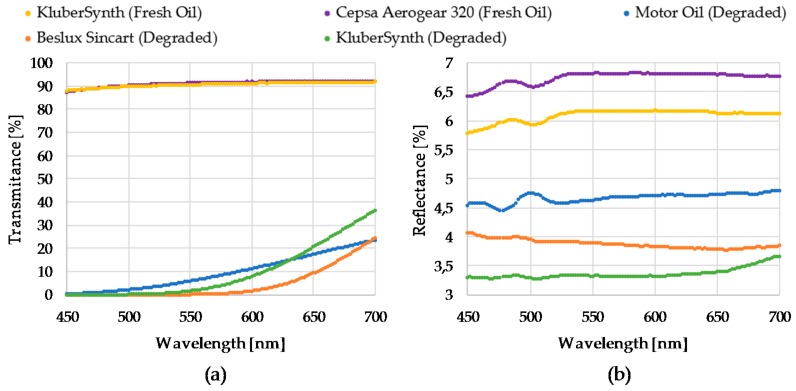
Graphical representation of the transmittance and reflectance of real oil samples measured with a laboratory equipment.

**Figure 12:**
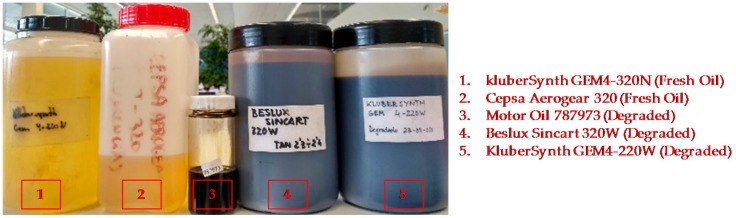
Real oil samples with different degradation status.

- The RGB colour sensor shows a sensibility of 30, 76 and 94 a.u. count/lx and the peak sensibilities 460 nm, 530 nm, 615 nm for Red, Green and Blue channels respectively, and directivity of 120° approximately.
- The feedback photodiode shows a 0.045 a.u. count/lx sensibility with a directivity of 90°.
- The white LED used as sample illuminator is able to deliver 2800 mcd at 30 mA driving current with a 120° 3 dB viewing angle. The emitter shows a relative luminous intensity of 90%, 65%, 80% at the peak sensibility wavelengths of the RGB photodetector.
- The construction and optical materials include: anodized aluminium for sensor body parts, which could be considered fully absorbent in the visible range, and optical back element of polished aluminium with an almost flat reflectance of 90% for visible spectra and BK7 optical window, which displays a transmittance of 99% also in the visible range.
- The light absorption at the oil sample will depend on its absorptivity (ε) and on the sample cavity length (L). Additionally, the total diffuse reflectance is also a parameter dependent on the chemical properties of the sample and on the sample length. Graphs displayed in Figure 11 show the transmittance and reflectance visible-NIR spectral values for different oil samples (see Figure 12) at different degradation status, measured with a laboratory equipment (LAMBDA 850 UV/Vis Spectrophotometer, Perkin Elmer, Waltham, MA, USA) using a 1 mm cuvette for the oil sample. According to these graphs and applying the Absorbance to Transmittance conversion displayed in equation (1), the absorbance of each sample can be obtained for the central wavelengths of the RGB colour sensor (see Table 5).

A = 2 − lg%T.(1)

According to measured transmittance (T [%]) and reflectance values (R [%]) (see Table 5), the selection of an optical back element of polished aluminium with an almost flat reflectance of 90% for visible spectra is a good option. It is observed that tested oils have a low reflectance capacity, so an optical back element with high and flat reflective capacity is required, such as the polished aluminum, that acts as a virtual light emitter (see Section 1.2) to increase the amount of light that could reach the RGB detector. 

Then, the Light Power Budget is calculated to show the relation between the light emitted by the LED and the light received by the RGB colour sensor, after passing through the different system elements (BK7 glass, oil sample, etc). As it is shown in Figure 11, with the aim of understanding the orders of magnitude of the photonic system and due to the complexity of the calculations, only the effect of direct light rays is considered in this theoretical approach. More detailed calculations will be presented in the simulation tests. The transmitted light intensity through the system elements is calculated according to the Beer-Lambert law, displayed in equation (2), where, I_0_ is the incident light intensity at wavelength λ, I is the transmitted intensity at same wavelength, ε is the molar absorption coefficient (L mol^–1^ cm^–1^) also called the molar extinction coefficient, c is the concentration (mol L^–1^) or Molar (M) and l is the light path along absorbing material (cm). 

To facilitate the calculation of transmitted light intensity, the next assumptions are considered:

- The absorbance coefficient of the air and BK7 glass is negligible.
- The refractive index of the BK7 glass and the fluid sample is negligible.
- The refractive index of the polished aluminium is 90%.
- The absorbances of the target oil for the defined cavity lengths (0.5, 1, 1.5, 2 mm) are calculated by applying the equation (2) to the values listed in Table 5. *KluberSynth* fresh oil and degraded samples are used, as they cover a complete degradation range.

(2) $$\;lg\frac{I_{o}}{I}=\varepsilon \cdot l\cdot c=A.$$

In this context, Table 6 shows the percentage of: (i) transmitted light intensity through the oil sample, (ii) reflected light intensity in the back element, and (iii) transmitted light intensity through the oil sample and received by the RGB colour sensor. Approximately measurement dynamic ranges of 60% and 80% are obtained with cavity lengths of 2 mm and 0.5 mm, respectively. The estimated luminous intensity that reaches the detector is considered enough to determine the feasibility proposed measurement principle setup. Many detailed calculations, considering directivity and sensibility peaks of the white LED and RGB colour sensor, will be performed in simulation tests in the next subsection.

#### 3.2.2. Optical Simulation of the Alpha Prototype (Activity 3 of the Activity Plan)

The objective of this simulation is to perform a more detailed analysis of the optical subsystem presented in the previous subsection. Different configurations of sample cavity length and fluid mediums are simulated to verify and optimize the position and characteristics of the proposed optical design components. In order to achieve reliable simulation results, all the optical subsystem components, including fluid sample, must be represented as real as possible. To obtain simulation results comparable to the experimental values, the different fluids of interest should be characterized across the entire visible spectrum in terms of absorbance, reflectance and transmittance. This characterization entails complex work that is not the objective of this study and could be carried out later if needed. Therefore, as it will be explained later in this subsection, standard materials will be used to simulate the fluid sample. As will be explained later, standard materials will be used to simulate the fluid sample. In this context, the simulation results do not provide a real value about the measurement dynamic range, but the best configuration and the feasibility of the presented optical design are analysed. The tool used to perform these simulations is ZEMAX OpticStudio^®^ (Zemax, Kirkland, WA, USA). 

First, the designed optical subsystem is represented. The white LED, the RGB colour sensor and the photodiode are represented following their commercial characteristics in terms of luminosity angle and expected spectral response. As described in the theoretical approach, the optical back element is simulated as a reflective (mirror) material. The 3 mm length anti-crosstalk shield and the material of the inner surface of the mechanical housing are considered absorbent components that collect all the light beams that impact on them. Finally, the BK7 glass optical window is included in the simulation model. Those elements are selected as ideal coatings/materials from the ZEMAX coating catalogue. 

Second, regarding the fluid sample simulation and taking into consideration the previously mentioned complexity of the fluid characterization, only the most extreme cases of 100% degraded and 0% degraded (fresh oil) fluid samples are simulated, as ideal absorbent and Lambertian scattering mediums, respectively. Thus, the absorbent medium is characterized as ideal absorbent material from the ZEMAX coating/material catalogue. The scattering of the light going through the fluid sample, is defined with a Lambertian scattering with a 0.55 ratio of scattered rays versus non-scattered rays. Most diffuse surfaces or volumes are nearly Lambertian, so it is a quite accurate model for our sample simulation. In the Lambertian scattering model, the scattered intensity is proportional to the cosine of the angle between the normal vector and the scattered ray angle. Lambertian scattering is independent of the ray incident angle. 

**Figure 13:**
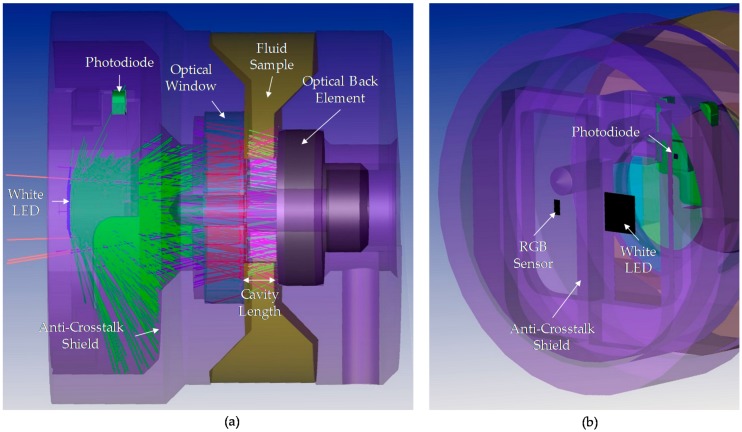
Lateral (**a**) and frontal (**b**) graphical representation of the light beam path inside the system for reflective (mirror) optical back element and anti-crosstalk shield configuration.

The simulation results are presented below. First, Figure 13 shows the path of light beam from emitter (white LED) to detector (RGB colour sensor) and to the photodiode (secondary receiver). The light rays are drawn up in different colours when they pass through the optical elements of subsystem (optical window, oil sample, etc.). Table 7 contains the lumens values in RGB colour sensor and photodiode for different sample cavity length and oil sample settings. To facilitate the interpretation of simulation results, it has been assumed that the emitter emits 1 Lumen/1.00 × 10^6^ rays.

As expected, taking into consideration the directivity of the main optical components (the white led, the RGB colour sensor, and the photodiode), and the absorbance and reflectance of the rest of the components, the RGB colour sensor receives less luminous intensity than that calculated in the theoretical approach (Section 3.2.1). However, the relation between the measurement of the absorbent and scattering samples verifies the feasibility of the proposed design. As mentioned before, although real fluid samples are not characterized, these simulation results suggest an approximately measurement dynamic range of 50%. It is considered that simulated ideal absorbent and Lambertian scattering media cover desired oil degradation states. In this context, a prototype with a configurable sample cavity length is developed to validate simulated optical subsystem’s functionality with real oil samples and to determine the real RGB measurement dynamic range. 

Finally, as the luminous intensity emitted by the white LED remains the same in all the simulations, the photodiode measurement is almost the same in all the cases. This result verifies that the position and characteristics of the photodiode are correctly selected.

#### 3.2.3. RGB Measurement Tests with Alpha Prototype (Activity 5 of the Activity Plan)

**Figure 14:**
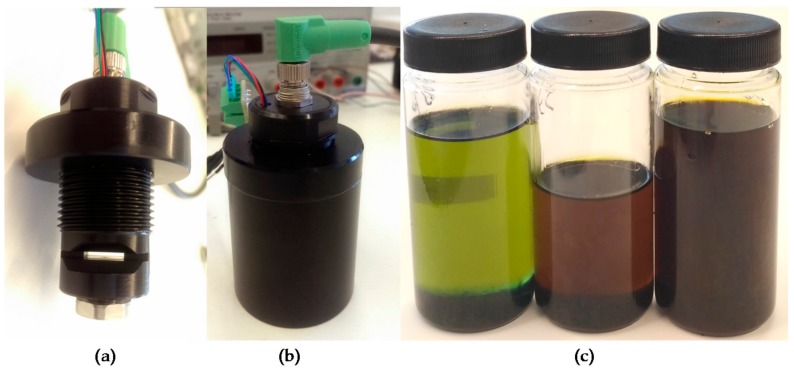
Developed (**a**) Alpha Prototype, (**b**) test bench, and (**c**) used oil samples (0%, 50% and 100% degraded) in the tests.

Based on the simulation result, a first prototype is developed to perform oil degradation validation tests with real samples (see Figure 14a). These tests determine the effectiveness of the system to measure the RGB absorbance of the oil sample and the RGB measurement dynamic range. This RGB measurement is used to calculate the oil degradation condition by applying embedded algorithms based on previously identified correlations between the physical-chemical variables of the lubricant oil and sample colour change [48].

To accomplish the tests, the prototype is introduced in the test bench shown in Figure 14b in order to prevent that external light from reaching the detector and to facilitate oil management. First, a measurement with the measurement cavity empty (no oil sample) is made to check prototype’s calibration and to ensure that the relationship between the light emitted by the white LED and the light received by the RGB colour sensor is correct. Based on the knowledge acquired in previous developments [53], it has been established that a correct calibration value corresponds to a measurement of 80% in the G channel. 

Next, measurements are made with samples of new and degraded oil of type *Agip OTE 32*. Figure 14c shows the oil samples used for these tests. These are samples of fresh oil (0% degraded) and 50% and 100% degraded oil (artificially degrades in a reactor). These samples were measured with different laboratory techniques including ASTM D664, D6971, E2412, D445, D1500 and D7843 and expertly diagnosed to obtain the real degradation value. Finally, these measurements are repeated for sample cavity lengths between 0.5 mm and 2 mm, adjusting the prototype’s reflective back element position. Obtained results are presented in Table 8.

**Figure 15:**
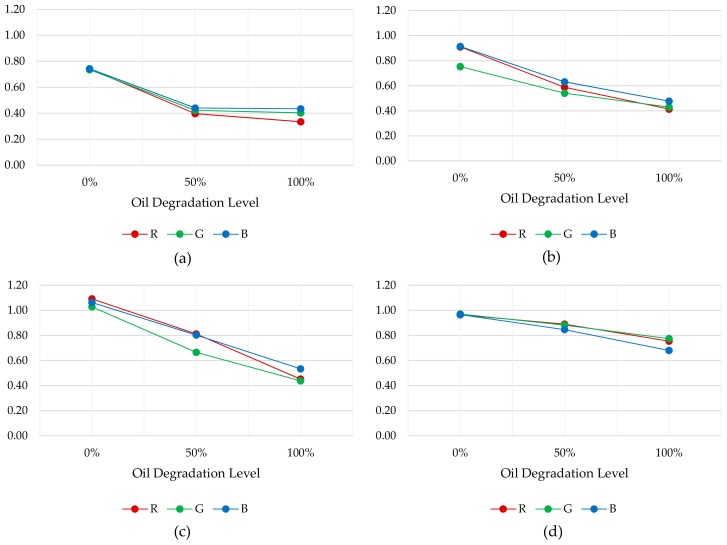
Graphical representation of the relationship between the measurements with oil sample and the empty measurement, for sample cavity lengths of (**a**) 2 mm; (**b**) 1.5 mm; (**c**) 1 mm; (**d**) 0.5 mm.

Tests results show that the difference between 100% and 0% degraded oil samples values for cavity lengths of 2 mm, 1.5 mm, 1 mm and 0.5 mm is 48%, 56%, 53% and 26%, respectively. These results determine that 2 mm, 1.5 mm and 1 mm lengths provide adequate measurement dynamic ranges. However, to analyse in detail the RGB measurement dynamic range for each sample cavity, the relationship between the oil sample measurements and the empty measurement is calculated. These results are illustrated in Figure 15. The graphs show that the widest and most linear measurement dynamic range is obtained with 1 mm sample cavity length. Consequently, it is assumed that it provides the best resolution to distinguish fresh and degraded oils. Besides, the 0.5 mm length provides a narrow and luminous measurement range, optimum for dark oil samples. On the other hand, 1.5 mm and 2 mm cavity lengths provides a narrow and dark measurement range, optimum for light oil samples. Consequently, the best configuration to cover the widest range of degradations and oil types is 1 mm length. Those results agree with the obtained simulations results, that determined a suitable sample cavity length of 1–2 mm. Those tests results support the design decision of setting a 1 mm sample cavity length.

#### 3.2.4. Optical Simulation of Beta Prototype (Activity 8 of the Activity Plan)

**Figure 16:**
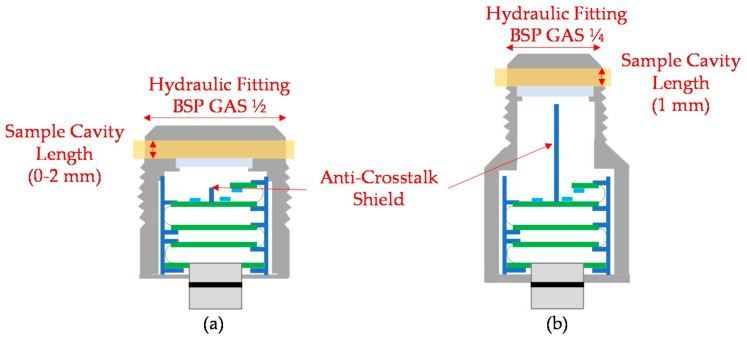
Block diagram of the Alpha (**a**) and Beta (**b**) sensor prototypes.

The objective of this simulation is to evaluate the impact that the increase in the distance between the transmitter and receiver and the fluid sample has on the other optical components and consequently on the RGB measurement. As mentioned before, this modification is motivated by a new customer mechanical specification that requires the reduction of the diameter of the hydraulic fitting (from BSP GAS ½ to BSP GAS ¼). In order to minimize the impact of this modification at to keep the investment in prototypes low, it is decided to move backwards the position of the sample cavity and keep the rest of the components as in Alpha Prototype. Figure 16 shows the main differences between Alpha and Beta prototypes.

This simulation is based on the previous simulation model and is performed as described in Section 3.2.2. First, according to previous test results, 1 mm sample cavity length is configured. Next, according to the new optical design, the sample cavity is set to a distance of 25.9 mm from the plane of light emitter and receiver. Finally, the characteristics of the anti-crosstalk shield and the inner surface of the mechanical housing are updated. To favour the number of light rays arriving at the detector, it is decided to simulate different configurations of anti-crosstalk shield lengths and inner surface of the mechanical housing. First, a configuration similar to the one used in the Alpha Prototype is selected. The anti-crosstalk shield is 3 mm length and of absorbent material. Next, this length is updated to 24 mm to cover almost all the distance between the emitter/receiver and the sample, to avoid the direct lighting between emitter and receiver. This shield configuration is simulated with absorbent and mirror materials. Finally, those three configurations are simulated with absorbent and reflective (mirror) materials for the inner surface of the mechanical housing. The mechanical housing of the Alpha Prototype was developed with an absorbent material (anodized aluminium), but it is expected that in this case a reflective (brass) configuration provides better results. As the sample is distanced from the light emitter and receiver, a reflective housing will favour the number of light rays arriving at the detector. 

**Figure 17:**
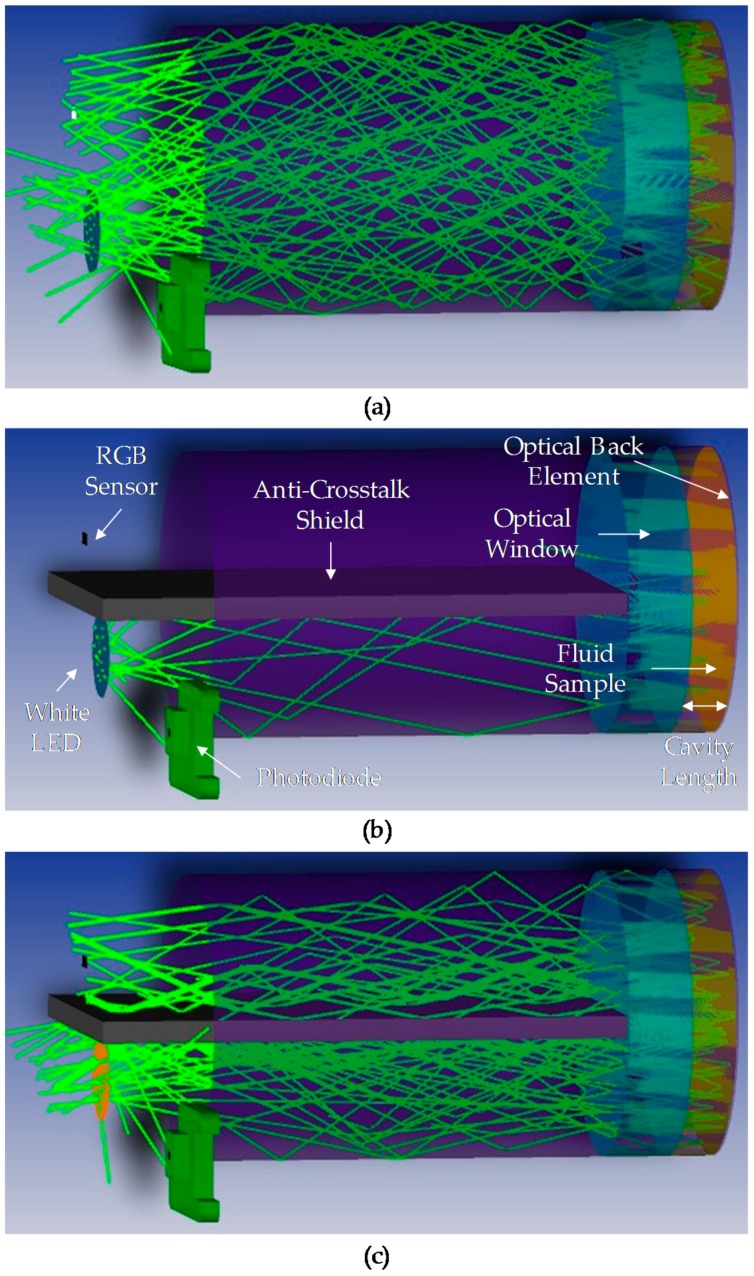
Graphical representation of the light beam path inside the system for reflective (mirror) inner surface of the mechanical housing and different anti-crosstalk shield configurations: (**a**) 3 mm absorbent anti-crosstalk shield; (**b**) 24 mm absorbent anti-crosstalk shield; (**c**) 24 mm reflective (mirror) anti-crosstalk shield.

In this context, Figure 17 shows the path of the light beam from the emitter to the detector simulating presented configurations. Moreover, Table 9 collects the lumen values in the RGB colour sensor and the photodiode for the defined simulation configurations. As in the previous simulation, to facilitate the interpretation of the simulation results, it has been assumed that the emitter emits 1 Lumen/1.00 × 10^6^ rays. 

The analysis of the simulation results (see Table 9 and Figure 17) determines that the configuration that optimized the optical design is the one corresponding to a reflective mechanical housing inner surface and a 24 mm reflective anti-crosstalk shield. Considering the rest of the configurations, the configuration with absorbent mechanical housing is discarded as the RGB detector barely receives luminous intensity. On the other hand, although a 3 mm anti-crosstalk shield maximizes the luminous intensity received by the RGB colour sensor receives, it is due to direct radiation from the emitter to the receiver, so this configuration is not suitable to provide a significant RGB measurement dynamic range. Finally, taking into consideration the simulation results of the 24 mm shield, the largest amount of luminous intensity is received with the reflective anti-crosstalk shield. Consequently, this is the optical subsystem design selected for the Beta Prototype. This design favours the propagation of the light rays due to the reflectivity of the inner surface of the mechanical housing and the anti-crosstalk shield and minimizes the incidence of direct light from the emitter to the RGB detector due to the anti-crosstalk shield length. As mentioned in the previous simulation, obtained results are not valuable to determine RGB measurement dynamic range as the real fluid samples are not characterized and ideal absorbent and Lambertian scattering mediums are simulated. In this context, a second prototype is developed to validate selected optical subsystem design with real oil samples and determine the real RGB measurement dynamic range. 

Finally, regarding the photodiode configuration, the simulation results are similar to the ones obtained in the Alpha Prototype simulation as this part of the design has not changed.

#### 3.2.5. RGB Measurement Tests with Beta Prototype (Activity 10 of the Activity Plan)

**Figure 18:**
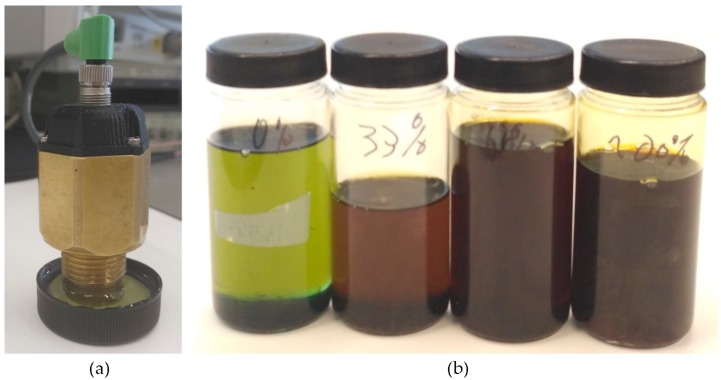
(**a**) Developed prototype and test-bench; (**b**) Oil samples (0%, 33%, 66% and 100% degraded) used in these tests.

Considering the success of the simulation results, the second prototype is developed. Again, this prototype is used to perform oil degradation measurement validation tests with real samples. As in Alpha Prototype, the test results determine the capacity of the new optical design to measure the RGB absorbance of the oil sample. The procedure used in these tests is the same than the one described in Section 3.2.3. First, to check optical calibration, a measurement with the measurement cavity empty (no oil sample) is performed. The G channel measurement must be around 80% to indicate a correct calibration. Next, measurements with different oil samples of *Agip OTE 32* type are taken. In order to extend the coverage of the tests, degraded samples are selected at 0% (fresh oil), 33%, 66% and 100% in this case (see Figure 18).

On the other hand, different configurations of the anti-crosstalk shield (absorbent and mirror) are used to check simulation results. In addition, these results are compared with measurements made with the Alpha Prototype and 1 mm sample cavity length. Table 10 collects obtained results. 

**Figure 19:**
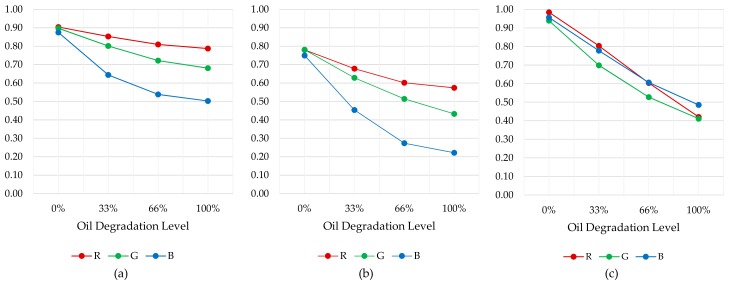
Graphical representation of the relationship between the measurements with oil sample and the empty measurement for: (**a**) Beta Prototype with absorbent anti-crosstalk shield; (**b**) Beta Prototype with reflective anti-crosstalk shield; (**c**) Alpha Prototype with 1mm sample cavity length and reflective back element.

Beta Prototype tests results show that the difference between 100% and 0% degraded oil samples values for 24 mm absorbent and reflective anti-crosstalk shield is 22% and 44%, respectively. As determined in simulation, the best optical configuration, and measurement dynamic range, is obtained with a reflective anti-crosstalk shield. On the other hand, taking into consideration Alpha Prototype (1 mm sample cavity length) measurements, the relationship between 100% and 0% degraded oil samples is 53%. In this context, comparable results are obtained with both prototypes. However, to analyse in detail the RGB measurement dynamic range, the relationship between the oil sample measurements and the empty measurement is also calculated. These results are illustrated in Figure 19. First, it is observed that the Beta Prototype R, G and B channel measurements (see Figure 19a,b) present a loss of linearity compared to the Alpha Prototype RGB measurements (see Figure 19c), over the entire oil degradation range. This is probably due to the change in the material of the mechanical housing. The Beta Prototype housing is made of brass, while Alpha Prototype housing is made of anodized aluminium. This modification was motivated by economic reasons, but it will be changed to polished aluminium in the final design due to the chromatic impact of the brass in the RGB measurement. Apart from that, the overall RGB measurement dynamic range of both the Alpha Prototype and the Beta Prototype with reflective anti-crosstalk shield is approximately 0.6. Those results support design decisions for the Beta Prototype and prove its feasibility. 

### 3.3. RGB Measurement Stability versus Temperature

This section describes the activities carried out to determine the dependence of the RGB measurement versus temperature (Alpha Prototype) and verify implemented lighting control (Beta Prototype) to compensate this effect and provide a stable measurement. 

#### 3.3.1. Theoretical Approach of Alpha Prototype versus Temperature (Activity 2 of the Activity Plan) 

This section describes the theoretical approximations made to determine the dependency of the main optical elements (white LED emitter and RGB colour) versus the temperature, and to establish a solution to compensate this effect.

**Figure 20:**
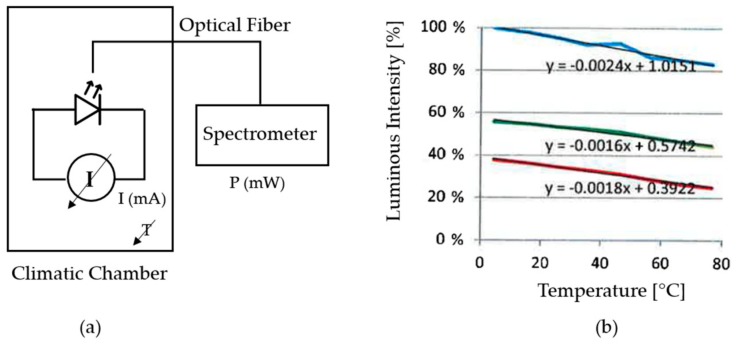
(**a**) Test setup; (**b**) Luminous intensity emitted by the white LED versus temperature, at the wavelengths of the colour blue, green and red.

**Figure 21:**
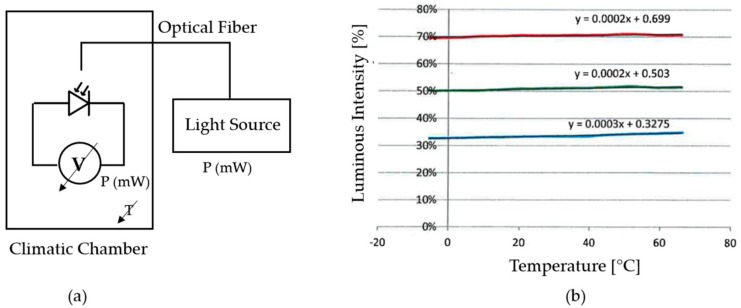
(**a**) Test setup; (**b**) Luminous intensity measured by the RGB colour sensor versus temperature.

- The dependency between the white LED and the temperature is tested making several measures with the LED in a climatic chamber over a temperature range of 0 °C to 80 °C. To measure the luminous intensity emitted by the LED, it is connected with an optical fiber to a VIS-NIR spectrophotometer (HR-2000+, Ocean Optics, Dunedin, FL, USA) placed outside the climatic chamber (see Figure 20a). Figure 20b shows measured luminous intensity at the wavelengths of the blue, green and red colours. A variation of approximately 20%, 13% and 15% is observed at wavelengths of the colour blue, green and red, respectively, over the tested temperature range.
- The dependency between the RGB colour sensor and the temperature is also tested making several measures with the RGB sensor in a climatic chamber over a temperature range of 0 °C to 80 °C. The RGB photodetector is illuminated with a 12 W broadband tungsten-halogen light source (HL-2000, Ocean Optics, Dunedin, FL, USA) placed outside the climatic chamber and connected to the colour sensor with an optical fiber (see Figure 21a). Figure 21 shows the measured luminous intensity at blue, green and red wavelengths. The variation of RGB measurement over the tested temperature range is negligible compared to the results obtained with the white LED.

Taking into consideration the white LED dependency versus temperature, it is necessary to implement lighting control to ensure light source stability over sensor’s operating temperature range. After a literature revision, the following lighting control approaches are identified:

- “Temperature Coefficient of Voltage Approach” measures the flow-through voltage of the white LED, which combined with the parameter Temperature Coefficient of Voltage allows sensing precisely the junction temperature of the LED, and based on this and the Temperature Coefficient of Efficiency, the referenced system implement an open loop control for maintaining the luminous intensity of the white LED constant [72].
- “Feedback Photodiode Approach” measures the luminous intensity emitted by the white LED with a secondary receiver, such as a photodiode, and based on this value an open control loop is implemented for maintaining the luminous intensity of the white LED constant [73].

The first option is discarded as it has a greater complexity of implementation and a custom calibration is required for each white LED to establish the relationship between desired luminous intensity and the flow-through voltage. The second approach does not require any calibration, as the luminous intensity emitted by the white LED is auto-calibrated and compensated with the photodiode measurement. However, it must be taken into consideration that the measurement of the selected photodiode has a variability of approximately 5% over the desired operation temperate range [73]. This variation is assumed versus the variation observed in the LED and considering the required RGB and OD measurements precision for the prototypes development.

#### 3.3.2. Temperature-Dependent RGB Measurement Stability Tests with Alpha Prototype (Activity 6 of the Activity Plan) 

**Figure 22:**
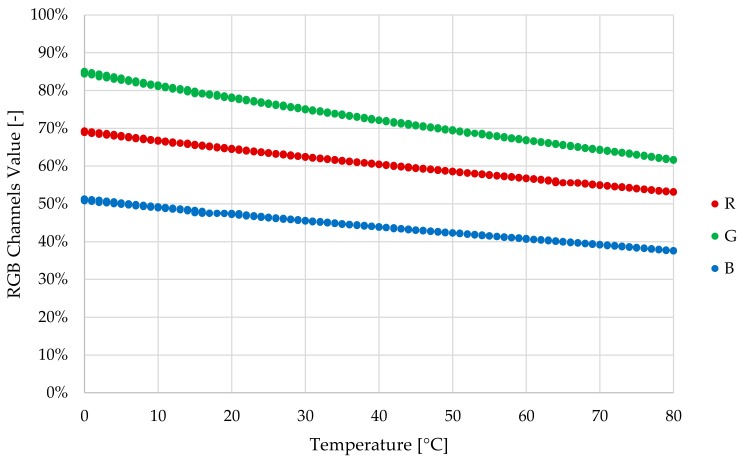
Graphical Representation of the RGB colour sensor measurement versus temperature, without lighting control in Alpha Prototype. The implemented algorithm automatically finds a unique luminance setpoint, which is the midpoint between the accepted minimum and maximum luminance values.

First, Alpha Prototype is used to perform RGB measurement stability tests in a climatic chamber. Measurements are made with no oil sample and 1 mm sample cavity length. Only this length is tested because the RGB absorbance measurement test results (see Section 3.2.3) determined the optimum measurement resolution at this length. The implemented temperature cycle is 0 °C–80 °C–0 °C in 6 h, making 1 measurement per minute. Tests results determine RGB measurement dependency versus temperature. As shown in Figure 22, RGB colour sensor measurement varies over the entire temperature range, approximately 20% in the G channel, 15% in the R channel and 10% in the B channel. Those results agree with the values determined in the theoretical approach, taking into consideration that, as described in the previous theoretical approach, relative luminous intensity peaks of the white LED do not match with the RGB colour relative sensitivity peak. However, observed RGB measurement variability is unacceptable, as it could lead to an erroneous interpretation of the oil degradation level provided by the sensor prototype. As mentioned in a previous subsection, the observed variability is mainly caused by the loss of efficiency of the white LED with. In this context, it is decided to implement a lighting control based on the photodiode measurement, to compensate this situation and provide a stable lighting, and consequently a stable RGB measurement. 

#### 3.3.3. Lighting Control Design and Simulation (Activity 9 of the Activity Plan)

**Figure 23:**
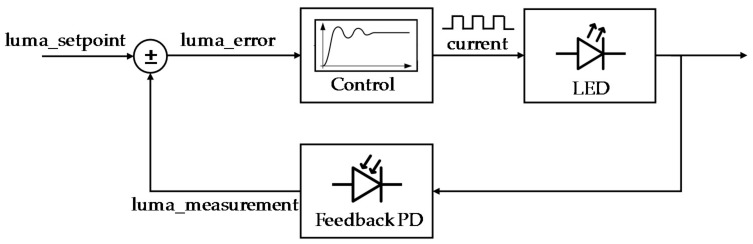
General block diagram of the lighting control implemented in Beta Prototype.

Figure 23 shows designed lighting control to compensate temperature effect in the white LED efficiency, and consequently the RGB measurement. The objective of the implemented control is to maintain an optimum luminance value (luma_setpoint_) over the entire working temperature range. White LED current is controlled by a pulse train, and the luminance value obtained for each pulse train configuration is measured by the photodiode (secondary receiver). 

Designed lighting control is a purely proportional control, in which the luminance error (the difference between luma_setpoint_ and luma_measurement_) is multiplied by an adjustable gain. The result is added to the LED train pulse value to obtain a new LED train pulse value to compensate the temperature effect.

**Figure 24:**
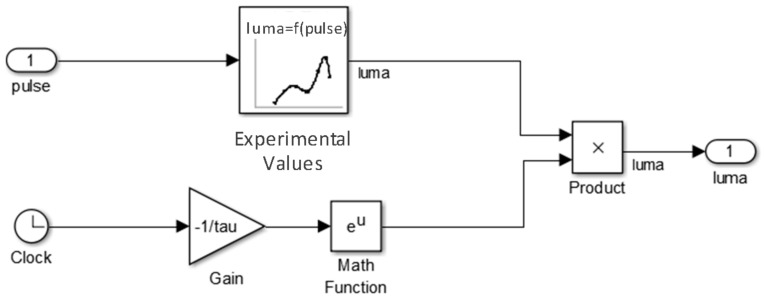
Diagram of the white LED model.

The lighting control is simulated and implemented using the MATLAB (Natick, MA, USA) computing environment. For the simulation, a model of the white LED is generated from experimental measurements. Those experimental values are linearly interpolated to obtain the white LED response for any LED pulse value. To emulate the white LED degradation or power loss versus temperature, an exponential decay over time is considered. Figure 24 shows the implemented white LED model. 

Furthermore, regarding lighting control implementation, instead of a single luminance setpoint an acceptable luminance range (luma_min_, luma_max_) is defined. The implemented algorithm automatically finds a unique luminance setpoint, which is the midpoint between the accepted minimum and maximum luminance values. 

**Figure 25:**
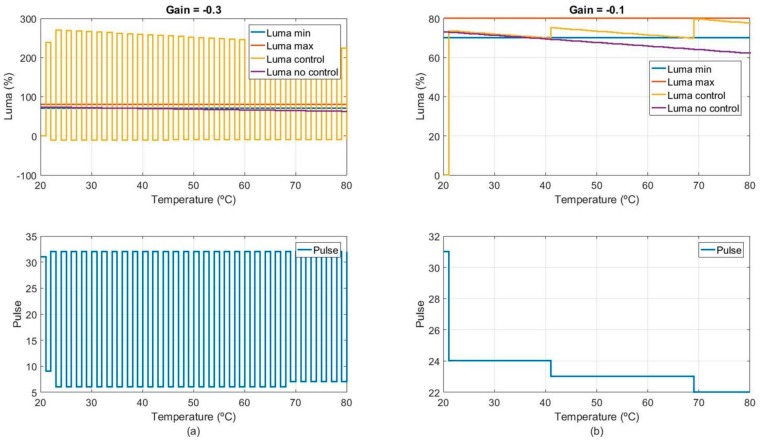
Graphical representation of simulated luminance and LED pulse values for different lighting control gain values ((**a**) −0.3; (**b**) −0.1).

Finally, Figure 25 shows simulation results for different gain value configurations. It is observed that from a certain gain value, the systems start to oscillate, and therefore the luminance value is not properly controlled (see Figure 25a). On the other hand, if an appropriate gain is selected, the system does not oscillate, and the control is correctly performed. However, in this case, Figure 25b shows that although the control gain is correct, the luminance is still outside the desired range. This is mainly due to the lack of resolution of the control hardware components, which does not allow to regulate the pulse value with greater precision. In this context, the minimum possible pulse variation will imply a luminance variation greater than needed. The only possible solution to this limitation is the redesign of the custom embedded electronic. At this point, it is decided that the current resolution may be valid so RGB measurement stability tests are performed with current custom embedded electronic design. However, the conclusions drawn from this simulation will be taken into consideration for future redesigns and sensor prototype updates. 

#### 3.3.4. Temperature-Dependent RGB Measurement Tests with Beta Prototype (Activity 11 of the Activity Plan) 

**Figure 26:**
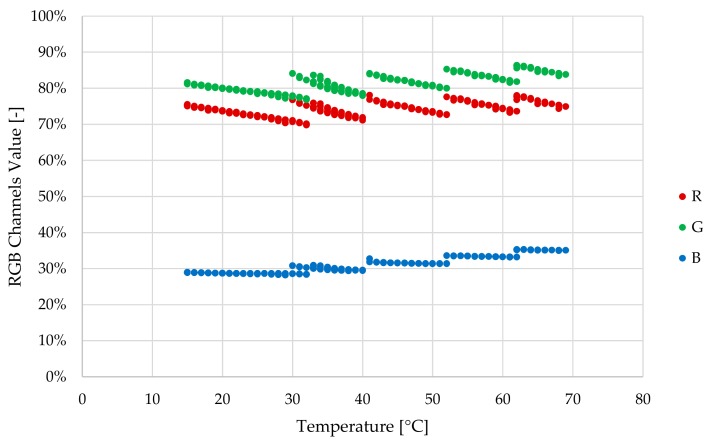
Graphical Representation of the RGB colour sensor measurement versus temperature, with the lighting control implemented in Beta Prototype.

Similar to previous temperature tests, the lighting control implemented in Beta Prototype is tested in the climatic chamber. The programmed temperature cycle is 0 °C–80 °C–0 °C in 6 h, making 1 measurement per minute. Figure 26 shows RGB colour sensor measurements. Those results determine that the total RGB measurement variability has decreased by around 50% compared to the results obtained with the Alpha prototype. Figure 26 shows that the RGB measurement variability of the Beta Prototype and the implemented lighting is around 10% for the G channel, 8 % for the R channel and 7% for the B channel. 

### 3.4. Fluidic Sample Acquisition: Sample Cavity Filling, Air Evacuation and Sample Renewal

This subsection presents the activities carried out to solve the technical, functional and design uncertainties of the fluidical-mechanical subsystem related to the fluidic sample acquisition (filling of the fluidic sample cavity, air evacuation and the renewal of the sample). As mentioned before, the presence of bubbles in the sample cavity, non-renewal of the oil sample, etc., can lead to an erroneous oil quality value. Performed activities to solve those uncertainties include several simulations to verify the proposed fluidical-mechanical design. These simulations will determine the pressure conditions of the hydraulic circuit in which the sensor will be installed to guarantee both the filling and the renewal of the fluidic sample. Then, fluidic tests are performed with Alpha Prototype to validate this design in almost real conditions.

The proposed fluidical-mechanical design is mainly conditioned by the requirements of the optical design (Section 3.2), and the integrability ease of installation in the hydraulic circuits without fluid recirculation.

#### 3.4.1. CFD Simulation of Alpha Prototype (Activity 4 of the Activity Plan)

The objective of the simulations described in this subsection is to determine the minimum flow rate that will pass through the sensor under a given pressure drop. To this end, simulations will verify sample cavity geometry and length, and the pressure conditions of the hydraulic lubricating circuit in which the sensor will be installed. This configuration will guarantee the fluid flow though the sensor, and consequently the filling of the sample cavity and oil sample renewal.

The simulation tool used to perform the next simulations is ANSYS Fluent CFD software (ANSYS, Canonsburg, PA, USA). In addition, the simulations are performed on the most extreme positions of estimated sensor sample cavity length: minimum position of 0.5 mm and maximum position of 2 mm. 

**Figure 27:**
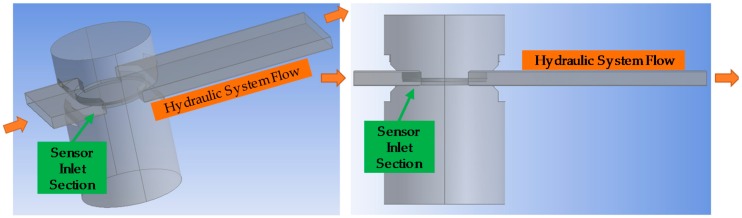
Different perspectives of the simulated geometry of the fluidical-mechanical part of the sensor, showing the expected flow through the sensor and fluid inlet and outlet sections.

As a first step, the geometry of the fluidical-mechanical part of the sensor is simulated to verify the inlet/outlet dimensions of the fluid sample cavity. Figure 27 shows the geometry of the fluidical-mechanical part of the sensor and the simulated hydraulic system flow. The simulation results show that the fluid in the sensor inlet section is organized and there are no whirlpools that could generate reverse flow. Thus, designed sensor’s fluid sample cavity geometry provides adequate fluid inlet and outlet.

Then, the steps taken to simulate the fluid flow through the sensor are described. This simulation provides the flow rate and pressure difference generated between the sensor inlet and outlet for a given sensor sample cavity length and pressure drop in the hydraulic installation circuit.

**Figure 28:**
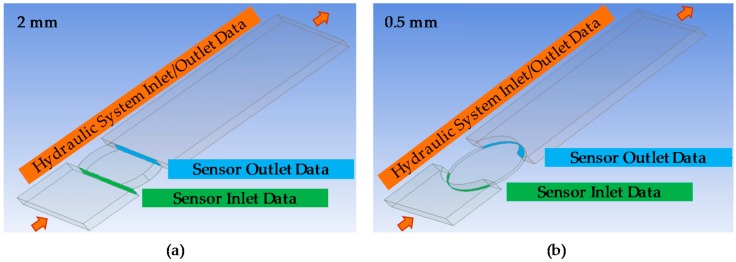
Defined liquid volume for the sample cavity lengths of (**a**) 2 mm and (**b**) 0.5 mm, indicating the simulation data collection points at the fluid inlet and outlet of both the hydraulic system and the sensor.

First, using the ANSYS DesignModeler toolbox (ANSYS, Canonsburg, PA, USA) of the simulation tool, liquid volume on which the simulations will be performed is defined. Figure 28 shows the defined liquid volume, considering sample cavity lengths of 2 mm and 0.5 mm. The difference in the shape of the defined liquid volume, corresponding to the inlet and outlet sensor sections, is due to the circularity of the optical back element and that it must be adjusted to reduce the sample cavity length thus modifying the boundary conditions. In addition, this figure shows the points at which the sensor and the hydraulic system input and output data will be taken for each of the simulations carried out below.

Second, to simulate the characteristics of the fluid, a meshing process has been performed with the ANSYS Meshing toolbox (ANSYS, Canonsburg, PA, USA). The Multizone method has been used and a limit layer has been imposed through Inflation. Regarding the mesh metric spectrum, Orthogonal Quality and Skewness has been selected.

Third, the pressure drop in the fluid has been calculated. To carry out this calculation, a Realizable K-epsilon Viscous Model with Enhanced Wall Treatment has been selected. In addition, the fluid that will pass through sensor has been created. Selected density and viscosity properties of fluid correspond to *Mobilgear-shc-xmp-320* lubricating oil (Density = 860 kg/m^3^ and Viscosity = 0.335 kg/m s).

Next, a simulation is performed to determine compliance with the mass balance and to calculate the mass flow generated through the sensor. As a boundary condition, a pressure loss ranging from 100 Pa (1 mbar) to 10000 Pa (0.1 bar) is imposed. Table 11 shows the results obtained in this simulation. It is observed that in all cases the mass balance is fulfilled, that a mass flow is generated, and that a mass flow corresponding to a laminar flow is generated. 

**Figure 29:**
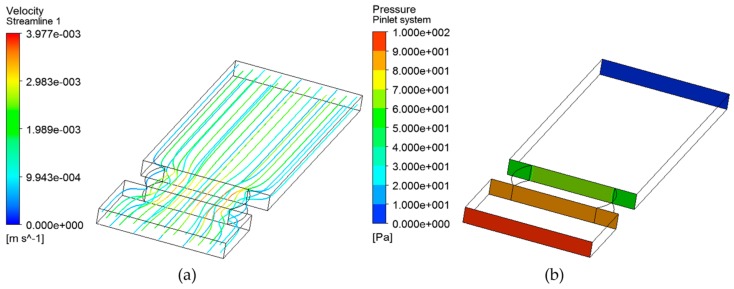
Representation of the flow velocity through the sensor (**a**) the pressure at the sensor and the hydraulic system inlet and outlet sections (**b**) for a hydraulic system pressure difference of 100Pa and a sample cavity length of 2 mm.

Finally, a post-processing of the results obtained in the previous simulation is performed. This post-processing determines the volumetric flow rate, the fluid speed along the sensor and the pressure difference between the sensor inlet and outlet. Figure 29 shows an example of these post-processing. The graphs show the flow velocity through the sensor and the pressure at the sensor and the hydraulic system inlet and outlet sections for the hydraulic system pressure difference of 100 Pa and a sample cavity length of 2 mm. Lastly, Table 11 shows all the information generated in this post-processing, regarding the different configurations of the sample cavity length and system pressure drop.

In conclusion, although the system pressure difference is low (100 Pa), simulation results confirm that the mass and volumetric flow through the sensor is guaranteed for any of the proposed fluidical­-mechanical design configurations (0.5 mm to 2 mm) and given fluid properties. Consequently, a pressure difference is generated in the sensor that will guarantee the filling of the measurement cavity, air evacuation and the renewal of the sample. These results confirm the viability of the proposed design provided that the hydraulic system in which the sensor is installed ensures at least this pressure drop and, therefore, the feasibility to continue with the development. However, although in oil storage tanks this pressure drop could not be guaranteed, diffusion principles will ensure sample renovation [74,75]. 

#### 3.4.2. Fluidic (Sample Acquisition) Test with Alpha Prototype (Activity 7 of the Activity Plan)

Based on the fluidic design proposed in the previous simulations, a prototype has been developed to validate the filling of the measurement cavity and the sample renewal under laboratory conditions. Considering that the validation of the optical design (Section 3.2) has determined that the best RGB absorbance measurement resolution is obtained with a sample cavity length of 1mm, the fluidic validation is only performed at this sample cavity length.

**Figure 30:**
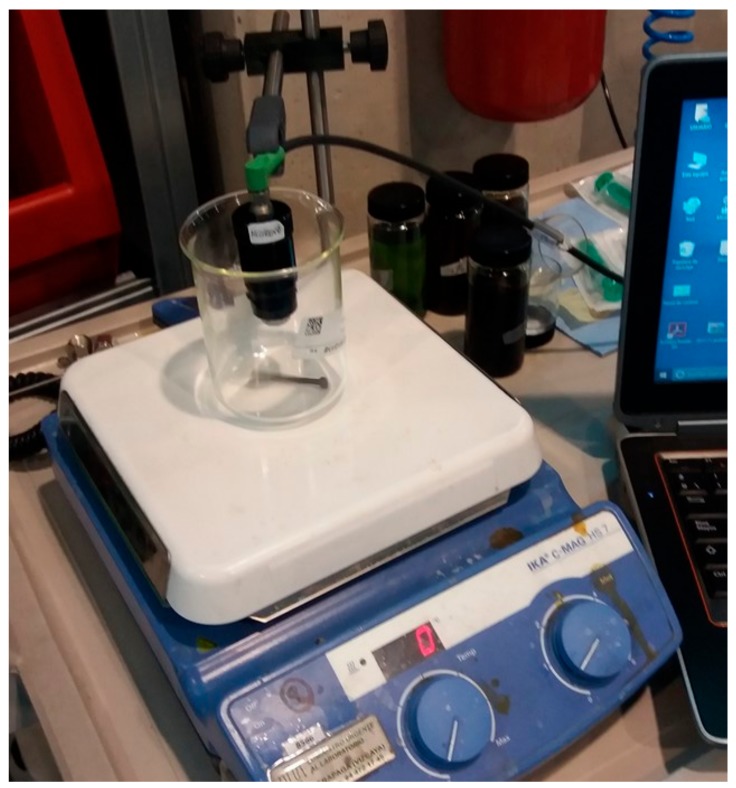
The test bench used to validate the filling of the measurement cavity and the renewal of the oil sample.

To perform this test, the prototype was immersed in 150 mL of new *Agip OTE 32* oil, and 5 mL of 100% degraded oil (artificially degraded in a reactor) was added at intervals of 5 min for 10 steps. To ensure oil mixing and sample renewal in the sensor, a magnetic stirrer with minimum stirring (1 rpm) was used. The sensor was then left to measure for another half hour to observe the evolution of the measurement. The test bench used to perform this test is shown in Figure 30. 

**Figure 31:**
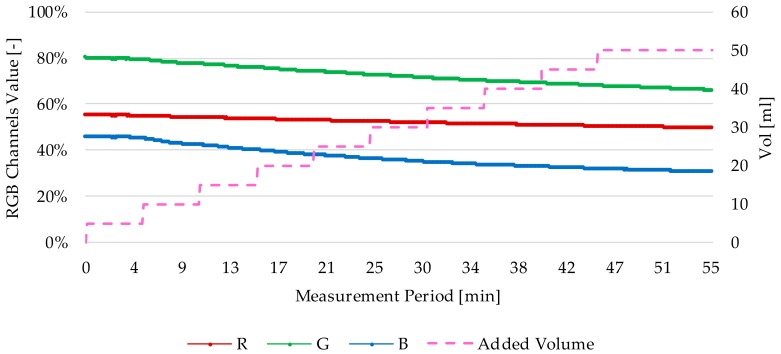
Graphical representation of the obtained RGB measurements in the fluidic sample cavity filling and renewal tests, against the added volume of 100% degraded oil sample.

On the other hand, Figure 31 shows the results obtained in the test. The graph shows the RGB absorbance value measured in different time periods (min.), compared to the volume of degraded oil added to the mixture. It is observed that as the degradation level of the mixture increases, the RGB measurement decreases. In addition, it is noticeable that there is a tendency to the stability in the RGB measurement during the final stabilization period of the mixture. This behaviour demonstrates that both the filling of sample cavity and the renewal of fluid sample are being carried out successfully.

## 4. Results and Discussion

Obtained conclusions from the development process are used to the develop a sensor product that meets all the requirements specified in terms of feasible measurement and operation, integrability, compact size and economic cost. Next, the compliance with those requirements is described in detail.

First, Table 12 present identified uncertainties at the beginning of the developments versus obtained results. Those results show that the expected performance related to oil degradation condition measurement resolution and feasibility, and sensor’s ease of installation and integrability are achieved. 

In addition, the additional requirements regarding compact size, economic cost and providing extra information about oil degradation condition are also met. On the one hand, the sensor size is Ø32 × 65 mm which is approximately the same as other similar commercial sensors (see Table 2). On the other hand, the sensor price is expected to be lower than the price range of the current commercial sensors (see Table 2). In this context, Table 13 shows expected manufacturing costs for the proposed sensor. Finally, Table 14 shows the results of the additional oil degradation condition information versus laboratory results. This information is obtained by applying implemented embedded algorithms to the RGB measurements of the Beta Prototype tests (Section 3.2.5). The difference between laboratory and sensor results fall within the range of ±5% for the oil degradation total index (OD) and ±10% for the rest of the parameters, which is an acceptable precision for in-line oil conditioning commercial sensors.

## 5. Conclusions

In this manuscript, a detailed description of the development process of an in-line oil condition sensor is presented. The sensor is based on an innovative setting form implementing a traditional measurement principle that determines the oil degradation condition from the change in colour of the sample over ageing time. The colour is measured using different light-matter interaction processes, such as transmittance and diffuse reflectance. In addition, the sensor aims to operate immersed in fluid to avoid oil sample conditioning hydraulic subsystems and consequently reduce sensor cost and size. This fact facilitates the integration and installation of the sensor in systems and machinery not specifically prepared and with reduced space for sensing. Finally, its low cost makes this sensor perfect for scenarios such as manufacturing plants where thousands of cranes and robotics arms should be monitored.

The development of the proposed sensor concept approach entails functional, technical and design uncertainties focused on the optical and fluidical-mechanical subsystems. On the one hand, the optical design must guarantee a significant and stable RGB measurement dynamic range to precisely measure required oil degradation conditions (colour changes). On the other hand, the fluidical-mechanical design must guarantee an adequate oil sample acquisition, including sample cavity filling, air evacuation and oil renewal. A failure at any of these points can lead to an erroneous oil degradation condition measurement and unreliable operation mode.

To successfully face and solve those uncertainties, a methodological development process based on NPD (New Product Development) approaches is followed. The process is based on theoretical approaches, simulations and prototypes development as intermediate solutions to efficiently reach a completely defined final sensor system. This process has led us to achieve a sensor of Ø32 × 65 mm and approximately cost of 100€/1000units (see Table 13), which is lower than the size and prize of similar commercial sensors. The sensor operates directly immersed in fluid if there is at least a 100 Pa pressure drop or, in scenarios such as oil storage tanks, Fick’s law of diffusion is met. In addition, the oil degradation condition information provided by the sensor includes a total oil degradation index (OD) and RULER, AN, FTIR (Oxi) and MPC (CLIELAB) parameters. Finally, the precision/variability of the measurement is ±5% for the OD and ±10% for the rest of the parameters over operation conditions, which is acceptable by the sensor client.

However, several improvements are identified. On the one hand, the emergence of new multifunctional sensor solutions, such as the HAMAMATSU Color/Proximity Sensor (Hamamatsu Photonics, Hamamatsu City, Japan) [76], could further reduce sensor size and improve measurement resolution/precision. Those multifunctional sensors incorporate into a single package an LED and a color sensor and improve emitted/received light ratio (photosensitivity) compared to the current design. On the other hand, migrating the lighting control to a “Temperature Coefficient of Voltage Approach” (see Section 3.3.1) or selecting a photodiode with a better temperature response could also improve measurement resolution/precision by reducing measurement variability over operating temperature conditions. The consideration of those new approaches in future work will improve the sensor performance quality. 

## 6. Patents

Mabe, J.; López, P.; Delgado, A.; Iturbe, I. (2017). Sistema y Método de Monitorización del Estado de un Fluido (System and Method for Monitoring the Condition of a Fluid). Fundación Tekniker. Spanish Patent and Trademark Office, Patent Application No. P201730848, June 2017.

## Figures and Tables

**Table 1:** Machinery that requires lubrication systems by sector and consequences associated with unscheduled shutdowns.

Sector		Oil (L)		Fault	Consequence
Electricity generation	Gas turbine	Turbine bearings (300 L)		Very serious	Machine standstill
	Steam turbine	Bearings (300 L)			Plant stop
	Wind turbine	Multiplier (300 L–1000 L)			Critical situation
Cement industry		Cement grinding reducer (2000 L)		Very serious	Line stop
		Other oil sumps (1000–1500 L)			
		Cement grinding supports (500 L)			
		Hydraulic group (80 L)			
Drainage and treatment of wastewater	Sanitation	Gas engine (>2000 L)		Very serious	Stop: 15–20 days
		Alternative hydraulic pump (800 L)		Less serious	Expensive breakdown
		Furnace pumps (500 L)			
	Treatment	Hydraulic turbines	Blade activation (400 L)	Serious/ Very serious	Stop: > a month
			Greasing generator bearings (400 L)		Expensive breakdown
Hydraulic presses and stamping	Hydraulic drawing machines	Small (3000 L)		Very serious	Stop
		Medium (3000 L–7000 L)			
		Large (7000 L–30,000 L)			
Steel sector		Forging press (>3.000 L)		Serious/ Very serious	Stop Expensive breakdown
		Drill press (1600 Tn)			
		Extortion press (3600 Tn)			
		Clamping presses (<600 L)			
		High pressure pumps (200 L–400 L)			
Automotive		Welding robot (1 L)		Very serious	Service deterioration, Unplanned outage
		Assembly robot (2 L)			
		Painting robot (1 L)			
Manufacturing		Hydraulic group (80 L)		Very serious	Plant unplanned outage, Expensive breakdown
		Process gearbox (40 L)			
		Process pumps (20 L)			
Storage tanks		Oils (100 KL)		Serious/ Very serious	Fluid disposal

**Table 2:** Commercial in-line sensors for lubricant status monitoring.

Sensor	Manufacturer	Measurement Principle	Information	Price	Size (mm)
LubCos	ARGO-HYTOS	Dielectric	Oil Condition, Temperature, Humidity	++	Ø42 × 147
OilQSens	b2 electronic gmbh	Permittivity Dielectric	Conductivity, Permittivity, Temperature, Tan Delta, Water, Breakdown Voltage	++++	Ø70 × 103
TRIDENT QW3100	Poseidon Systems	Electrochemical impedance spectroscopy	Additive, contaminants, water	++	Ø38 × 121
ANALEXrs Oil condition	Kittiwake	Dielectric constant	Oil Condition (%)	++	Ø30 × 130
Oil Condition Sensor	Lubrigard Ltd.	Dielectric loss factor	Oil contamination	++++	Ø37 × 76
OQSx	TANDeltaSystems	Tan Delta	Oil Quality (Tan Delta Number) Oil Temperature	++	Ø37 × 90
Oil Insyte	Voelker Sensors Inc.	Dielectric Constant	Oxi	++	Ø35 × 120
Oil Quality Sensor OQS	STAUFF	Dielectric	Oil Quality	++++	Ø37 × 80

**Table 3:** Description of the identified functional, technical and design uncertainties of the proposed sensor concept approach and possible solution mechanisms.

		Solution Mechanisms
Functional	Technical/Design	
Significant RGB measurement	Position and characteristics of the optical elements (light source, RGB detector, optical back element, etc.) to maximize RGB measurement dynamic range (Optical Subsystem Design).	▪ Theoretical approach ▪ Simulation ▪ Prototypes development and tests
	Sample cavity design to guarantee a correct sample acquisition: cavity filling, air evacuation and fluid sample renewal (Fluidical-Mechanical Subsystem Design).	▪ Simulation ▪ Prototypes development and tests
Stable RGB measurement	Dependency between the light source variability versus temperature and the RGB measurement variability, and required stability control mechanisms (Optical Subsystem Design).	▪ Theoretical approach ▪ Simulation ▪ Prototypes development and tests

**Table 4:** Relationship between the activities defined in the development process plan and the structure of this section.

Section	Activity	Uncertainty
3.2.1	1. Theoretical Approach of the Optical Subsystem of the Alpha Prototype	RGB Measurement Dynamic Range
3.2.2	3. Optical Simulation of the Alpha Prototype	
3.2.3	5. RGB Measurement Tests with Alta Prototype	
3.2.4	8. Optical Simulation of the Beta Prototype	
3.2.5	10. RGB Measurement Tests with Beta Prototype	
3.3.1	2. Theoretical Approach of the Optical Subsystem of the Alpha Prototype versus Temperature	RGB Measurement Stability
3.3.2	6. Temperature-Dependent RGB Measurement Tests with Alpha Prototype	
3.3.3	9. Lighting Control Design and Simulation	
3.3.4	11. Temperature-Dependent RGB Measurement Tests with Beta Prototype	
3.4.1	4. Computational Fluidic Dynamic (CFD) Simulation of the Alpha Prototype	Fluidic Sample Acquisition
3.4.2	7. Fluidic (Sample Acquisition) Tests with Alpha Prototype	

**Table 5:** Transmittance, reflectance and absorbance values of each oil sample for the central wavelengths of the RGB sensor.

Oil Sample	λ = 460 nm (Blue Colour)			λ = 530 nm (Green Colour)			λ = 614 nm–616 nm (Red Colour)		
	T [%]	R [%]	A	T [%]	R [%]	A	T [%]	R [%]	A
Kluber (Fresh)	88.36	5.84	0.0534	90.35	6.14	0.0437	91.26 91.29	6.16 6.17	0.0393 0.0393
Cepsa (Fresh)	88.27	6.46	0.054	90.98	6.8	0.0409	91.91 91.89	6.81 6.8	0.0366 0.0368
Motor (Degraded)	0.58	4.59	2.2363	4.16	4.59	1.3799	12.95 13.19	4.72 4.71	0.8868 0.879
Beslux (Degraded)	0	4.02	4.9848	0.02	3.91	3.7486	3.01 3.23	3.81 3.81	1.5348 1.5019
Kluber (Degraded)	0	3.29	5.0236	0.66	3.33	2.1821	10.88 11.34	3.34 3.34	0.9636 0.9455

**Table 6:** Transmitted/Reflected light intensity through different elements of the proposed measurement principle setup.

Cavity Length	(i) Oil Sample (Transmitted Light)			(ii) Back Element (Reflected Light)			(iii) Oil Sample (Transmitted Light) → RGB Detector		
	B	G	R	B	G	R	B	G	R
	*KluberSynth fresh oil*								
0.5 mm	94%	95%	96%	85%	86%	86%	80%	81%	82%
1 mm	88%	90%	91%	80%	81%	82%	70%	74%	75%
1.5 mm	83%	86%	87%	75%	77%	79%	62%	67%	69%
2 mm	78%	82%	83%	70%	74%	75%	55%	60%	63%
	*KluberSynth degraded oil*								
0.5 mm	0%	8%	33%	0%	7%	30%	0%	1%	10%
1 mm	0%	1%	11%	0%	1%	1%	0%	0%	1%
1.5 mm	0%	0%	4%	0%	0%	3%	0%	0%	0%
2 mm	0%	0%	1%	0%	0%	1%	0%	0%	0%

**Table 7:** Simulation results: lumens in the RGB colour sensor and the photodiode for different configurations of sample cavity length and oil samples.

Oil Sample	Absorbent			Scattering		
Cavity Length	0.5 mm	1 mm	2 mm	0.5 mm	1 mm	2 mm
RGB (Lumens)	3.98 × 10^−6^	3.26 × 10^−6^	3.14 × 10^−6^	6.92 × 10^−6^	7.09 × 10^−6^	7.59 × 10^−6^
Photodiode (Lumens)	2.52 × 10^−4^	2.61 × 10^−4^	2.55 × 10^−4^	2.60 × 10^−4^	2.59 × 10^−4^	2.64 × 10^−4^
Emitter: 1 Lumen/1.00 × 10^6^ rays						

**Table 8:** Tests results: RGB measurements value for different oil samples and sample cavity length configurations.

Cavity Length	2 mm			1.5 mm			1 mm			0.5 mm		
Oil Sample	R	G	B	R	G	B	R	G	B	R	G	B
No sample	59%	82%	49%	55%	84%	48%	51%	88%	48%	63%	84%	46%
0% Degraded	44%	61%	36%	50%	63%	44%	56%	91%	51%	60%	82%	44%
50% Degraded	24%	35%	22%	32%	45%	30%	41%	59%	39%	56%	74%	34%
100% Degraded	20%	33%	21%	23%	36%	23%	23%	39%	26%	47%	65%	26%

**Table 9:** Simulation results: lumens in the RGB colour sensor and the photodiode for different configurations of inner surface of the mechanical housing, anti-crosstalk shield and oil sample.

Housing	Absorbent				Mirror			
Oil Sample	Absorbent		Scattering		Absorbent		Scattering	
Anti-Crosst Shield	RGB	PD	RGB	PD	RGB	PD	RGB	PD
3 mm (Absorbent)	7.93 × 10^−7^	2.56 × 10^−4^	1.26 × 10^−6^	2.62 × 10^−4^	3.76 × 10^−5^	2.64 × 10^−4^	5.98 × 10^−5^	2.52 × 10^−4^
24 mm (Absorbent)	0	2.57 × 10^−4^	3.19 × 10^−9^	2.68 × 10^−4^	0	2.64 × 10^−4^	6.02 × 10^−6^	2.55 × 10^−4^
24 mm (Mirror)	0	3.05 × 10^−4^	1.16 × 10^−8^	3.04 × 10^−4^	3.50 × 10^−8^	2.99 × 10^−5^	2.30 × 10^−5^	2.96 × 10^−4^
Emitter: 1 Lumen/1.00× 10^6^ rays.								

**Table 10:** The tests results: RGB measurements value of Beta and Alpha Prototypes (1 mm sample cavity length) for different oil samples, inner surface of the mechanical housing and anti-crosstalk shield configurations.

	Beta Prototype						Alpha Prototype		
Inner Housing	Reflective						Absorbent		
Anti-Crosstalk Shield	24 mm Absorbent			24 mm Reflective			3 mm Absorbent		
Oil Sample	R	G	B	R	G	B	R	G	B
No Sample	75%	81%	29%	61%	80%	41%	62%	81%	50%
0% Degraded	68%	72%	25%	48%	63%	31%	61%	76%	48%
33% Degraded	64%	65%	18%	41%	51%	18%	50%	56%	39%
66% Degraded	60%	58%	15%	37%	41%	11%	37%	43%	30%
100% Degraded	59%	55%	14%	35%	35%	9%	26%	33%	24%

**Table 11:** Fluidic simulation results.

ΔP System Input/Output [Pa]	Mass Balance	Mass Flow [kg/s]	Volumetric Flow [m^3^/s]	ΔP Sensor Input/Output [Pa]
Sample Cavity Length = 2 mm (V_sensor_ = 2.5336 × 10^−7^ m^3^)				
100 Pa	Satisfy	4.58 × 10^−5^	5.325962 × 10^−8^	17.07
1000 Pa	Satisfy	4.579 × 10^−4^	5.325 × 10^−7^	171.01
10000 Pa	Satisfy	4.579 × 10^−3^	5.3136 × 10^−6^	1706.35
Sample Cavity Length = 0.5 mm (V_sensor_ = 6.334 × 10^−8^ m^3^)				
100 Pa	Satisfy	2.8517 × 10^−6^	3.316 × 10^−5^	95.48
1000 Pa	Satisfy	2.8367 × 10^−5^	3.298 × 10^−5^	956.96
10000 Pa	Satisfy	2.8359 × 10^−4^	3.298 × 10^−7^	9569.14

**Table 12:** Identified initial uncertainties and obtained results.

Uncertainties		Obtained Results
Functional	Technical/Design	
Significant RGB measurement	Position and characteristics of the optical elements to maximize RGB measurement dynamic range (Optical Subsystem Design).	Significant/Representative RGB measurement dynamic range within tested 0% and 100% degraded real oil samples (see Section 3.2).
	Sample cavity design to guarantee a correct sample acquisition (Fluidical-Mechanical Subsystem Design).	Minimum pressure drops of 100 Pa or compliance with diffusion principles to ensure sample acquisition (see Section 3.4).
Stable RGB measurement	Measurement dependency versus temperature and required stability control mechanisms (Optical Subsystem Design).	10% deviation for channel G, 8% for channel R and 7% for channel B versus temperature (see Section 3.3).

**Table 13:** Approximate sensor manufacturing costs.

	Cost
Components	35 €
PCB	4 €
Mechanics	27 €
Assembly	(5 min) 5 €
Calibration	(30 min) 30 €
TOTAL	101 €

**Table 14:** Oil degradation condition information provided by the sensor versus laboratory results.

Oil Sample	OD	RULER	AN	FTIR (Oxi)	MPC (cielab)
Laboratory Results					
Fresh oil	0%	100%	0.18	<1	0
33% Degraded	33%	47%	0.22	<1	10
66% Degraded	66%	21%	0.35	2	25
100% Degraded	100%	8%	0.47	5	45
Sensor (Beta Prototype)					
33% Degraded	37%	44.12%	0.17	0	8
66% Degraded	68%	12.56%	0.29	2	23
100% Degraded	96%	1%	0.42	6	48
